# An investigation to offer conclusive recommendations on suitable benefit/cost criteria-based normalization methods for TOPSIS

**DOI:** 10.1016/j.mex.2023.102227

**Published:** 2023-05-30

**Authors:** Anath Rau Krishnan, Mohamad Rizal Hamid, Geoffrey Harvey Tanakinjal, Mohammad Fadhli Asli, Bonaventure Boniface, Mohd Fahmi Ghazali

**Affiliations:** a Labuan Faculty of International Finance, University Malaysia Sabah, Jalan Sg. Pagar, 87000, Labuan F.T., Malaysia; b Faculty of Computing and Informatics, University Malaysia Sabah, Jalan Sg. Pagar, 87000, Labuan F.T., Malaysia; c Centre for the Promotion of Knowledge and Language, Universiti Malaysia Sabah, Jalan UMS, 88400, Sabah, Malaysia; d Faculty of Economics and Management, Universiti Kebangsaan Malaysia, 43600 Bangi, Selangor, Malaysia

**Keywords:** Multi-criteria decision-making, Normalization, TOPSIS, TOPSIS

## Abstract

Technique for Order Preference by Similarity to Ideal Solution (TOPSIS) is a popular multi-criteria decision-making method that ranks the available alternatives by examining the ideal-positive and ideal-negative solutions for each decision criterion. The first step of using TOPSIS is to normalize the presence of incommensurable data in the decision matrix. There are several normalization methods, and the choice of these methods does affect TOPSIS results. As such, some efforts were made in the past to compare and recommend suitable normalization methods for TOPSIS. However, such studies merely compared a limited collection of normalization methods or used a noncomprehensive procedure to evaluate each method's suitability, leading to equivocal recommendations. This study, therefore, employed an alternate, comprehensive procedure to evaluate and recommend suitable benefit/cost criteria-based normalization methods for TOPSIS (out of ten methods extracted from past literature). The procedure was devised based on three evaluation metrics: the average Spearman's rank correlation, average Pearson correlation, and standard deviation metrics, combined with the Borda count technique.

• The first study examined the suitability of ten benefit/cost criteria-based normalization methods over TOPSIS.
• Users should combine the sum-based method and vector method into the TOPSIS application for safer decision-making.
• The maximum method (version I) or Jüttler's-Körth's method has an identical effect on TOPSIS results.

## Graphical abstract

**Figure:**
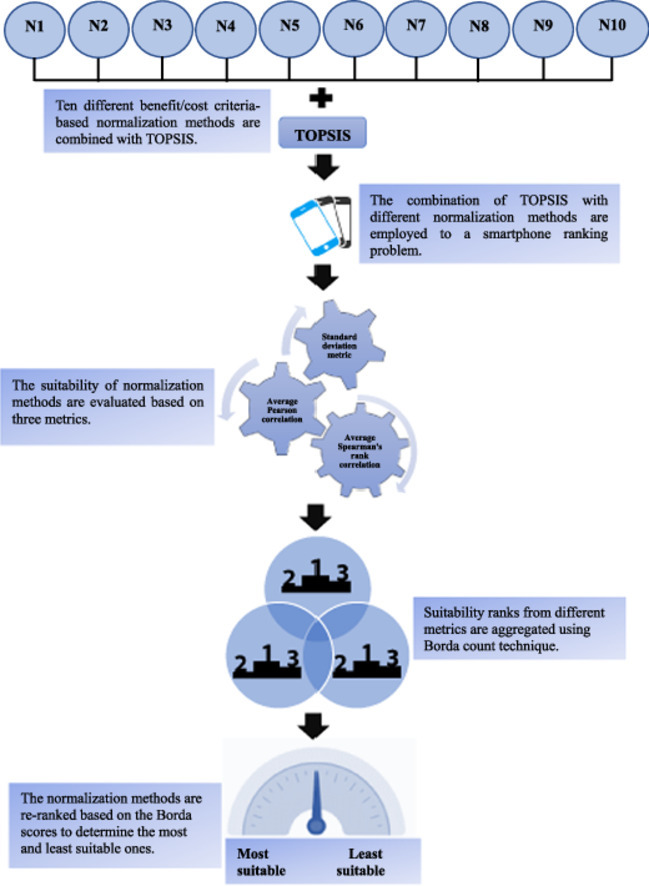

Specifications table

**Table:** 

Subject area:	Mathematics and Statistics
More specific subject area:	Multi-criteria decision-making
Name of your method:	TOPSIS
Name and reference of original method:	Hwang, C.L.; Yoon, K. (1981). Multiple Attribute Decision Making: Methods and Applications. New York: Springer-Verlag.
Resource availability:	Link for visualization, i.e., Fig. 2https://public.tableau.com/views/Figure2-Methodsx/Figure2-MethodsX?:language=en-US&publish=yes&:display_count=n&:origin=viz_share_link

## Introduction

A multi-criteria decision-making (MCDM) technique enables decision-makers to systematically evaluate and rank a finite group of alternatives based on various conflicting decision criteria [1]. A wide range of popular MCDM techniques are available for solving complex decision problems, including TOPSIS, the Analytic Hierarchy Process (AHP) and the Analytic Network Process (ANP).

However, the TOPSIS technique, originally developed by Hwang and Yoon in 1981 [2], holds some notable merits compared to AHP and ANP. For example, TOPSIS is relatively easy to understand and apply since it has a straightforward computational procedure and uses a simple yet logical principle in analyzing the preference over alternatives [3]. In contrast, AHP and ANP can be more complex and require greater expertise for the appropriate application, particularly when more criteria or alternatives are present [4]. Besides, AHP and ANP require pairwise comparisons between criteria or alternatives, which can be time-consuming [5] and challenging to maintain an acceptable degree of consistency in the comparisons made [6]. TOPSIS, on the other hand, does not require pairwise comparisons and hence is less demanding in the context of data collection and processing [7].

As such, more and more academic researchers and practitioners are magnetized to employ TOPSIS in dealing with complex decision problems. Indeed, in the past, the method was applied to MCDM problems that occurred in domains like water resources management [8], renewable energy [9], higher education [10], manufacturing [11], agriculture [12], pharmaceutical [13], to name a few. The use of TOPSIS has also been considered in the development of various recommender systems, such as the hotel recommender system [14], the sports venue recommender system [15], and the job training recommendation system [16].

The technique, in principle, identifies the positive-ideal and negative-ideal solutions for each decision criterion and ranks the alternatives by comparing their geometric distance from the positive-ideal and negative-ideal solutions [17]. The technique ensures the alternative with the shortest distance from the positive-ideal solution and the farthest distance from the negative-ideal solution tops the rank.

On the other hand, like many other MCDM techniques, the core input for TOPSIS is the decision matrix that encapsulates the data of alternatives across different decision criteria [18]. Assume an MCDM problem in which $A_{i}=\left\{A_{1},\;A_{2},\ldots ,\;A_{m}\right\}\;$  represents the alternatives under consideration and $C_{j}=\left\{C_{1},\;C_{2},\ldots ,\;C_{n}\right\}$  denotes the set of decision criteria. The decision matrix can then be expressed in a general form as (1), where $x_{mn}$ denotes the data value of alternative m over criterion n.

(1) $$\begin{array}{ccccc} Alternatives/criteria & C_{1} & C_{2} & \cdots & C_{n}\\ A_{1} & x_{11} & x_{12} & \cdots & x_{1n}\\ A_{2} & x_{21} & x_{22} & \ldots & x_{2n}\\ \vdots & \vdots & \vdots & \vdots & \vdots \\ A_{m} & x_{m1} & x_{m2} & \cdots & x_{mn} \end{array}$$

In reference to (1), the criteria in a decision matrix usually carry different units of measurement [19]. For instance, in a drone selection problem, maximum speed, camera quality, battery power, and price are measured in metres per second, megapixels, milliampere-hours, and dollars, respectively. As such, the data in the decision matrix should be converted into a commensurable unit to allow the decision-makers to make a logical data comparison across different criteria [20]. Indeed, the use of many MCDM techniques, including TOPSIS, begins with a procedure for transforming the data in the decision matrix into a standard unit. This procedure is widely known as normalization. The procedure eliminates unit differences and converts the data values across all criteria to a standard scale [21].

## Literature review and motivation of the study

There are numerous normalization methods available in the literature. These methods can generally be divided into two categories, i.e., the benefit/cost criteria (BCC)-based methods and non-BCC-based methods [22]. However, the scope of this study is limited to benefit/cost criteria (BCC)-based methods. It is because these methods appear as a more preferred choice for most of the MCDM problems since they consider whether a criterion is a benefit or cost criterion before normalization—the benefit criterion implies that the higher the value, the better, whereas the cost criterion means that the lower the value, the better [23,24]. As such, using a BCC-based method for an MCDM problem permits a logical comparison of an alternative across different criteria.

Having said that, it is known that associating different BCC-based normalization methods with an MCDM technique can deliver deviating results [25]. Therefore, choosing the ideal normalization methods for an MCDM technique has emerged as an intriguing topic of investigation. Various efforts were made in the past to compare the suitability of a normalization method, and Table 1 presents a list of such comparative studies and the BCC-based methods compared. The MCDM technique(s) considered in each study is (are) also presented in Table 1. The formulas for each method listed in Table 1, for both benefit and cost criteria, are denoted by (2) - (11), where $x_{ij}$ is the data value of alternativei with respect to criterion j, $r_{ij}$  is the normalized data value of alternative i over criterion j, $\text{ma}x_{j}\left(x_{ij}\right)$ is the maximum data value of criterion j, and $\text{mi}n_{j}\left(x_{ij}\right)$ is the minimum data value of criterion j.

(2) $$N1;\;\;\;r_{ij}=\left\{ \begin{array}{c} \frac{x_{ij}}{\sum_{i=1}^{m}x_{ij}},\;\;\;\text{for}\;a\;\text{benefit}\;\text{criterion}\\ \frac{1{/x}_{ij}}{\sum_{i=1}^{m}1{/x}_{ij}},\;\;\;\text{for}\;a\;\text{cost}\;\text{criterion} \end{array}\right.$$

(3) $$N2;\;\;\;r_{ij}=\left\{ \begin{array}{c} \frac{x_{ij}-{min}_{j}\left(x_{ij}\right)}{{max}_{j}\left(x_{ij}\right)-{min}_{j}\left(x_{ij}\right)},\;\;\;\text{for}\;a\;\text{benefit}\;\text{criterion}\\ \frac{{max}_{j}\left(x_{ij}\right)-x_{ij}}{{max}_{j}\left(x_{ij}\right)-{min}_{j}\left(x_{ij}\right)},\;\;\;\text{for}\;a\;\text{cost}\;\text{criterion} \end{array}\right.$$

(4) $$N3;\;\;\;r_{ij}=\left\{ \begin{array}{c} \frac{x_{ij}}{{max}_{j}\left(x_{ij}\right)},\;\;\;\text{for}\;a\;\text{benefit}\;\text{criterion}\\ 1-\frac{x_{ij}}{{max}_{j}\left(x_{ij}\right)},\;\;\;\text{for}\;a\;\text{cost}\;\text{criterion} \end{array}\right.$$

(5) $$N4;\;\;\;r_{ij}=\left\{ \begin{array}{c} \frac{x_{ij}}{{max}_{j}\left(x_{ij}\right)},\;\;\;\text{for}\;a\;\text{benefit}\;\text{criterion}\\ \frac{{min}_{j}\left(x_{ij}\right)}{x_{ij}},\;\;\;\text{for}\;a\;\text{cost}\;\text{criterion} \end{array}\right.$$

(6) $$N5;\;\;\;r_{ij}=\left\{ \begin{array}{c} \frac{x_{ij}}{{max}_{j}\left(x_{ij}\right)},\;\;\;\text{for}\;a\;\text{benefit}\;\text{criterion}\\ \frac{{x_{ij}-min}_{j}\left(x_{ij}\right)}{{max}_{j}\left(x_{ij}\right)},\;\;\;\text{for}\;a\;\text{cost}\;\text{criterion} \end{array}\right.$$

(7) $$N6;\;\;\;r_{ij}=\left\{ \begin{array}{c} \frac{x_{ij}}{\sqrt{\sum_{i=1}^{m}x_{ij}^{2}}},\;\;\;\text{for}\;a\;\text{benefit}\;\text{criterion}\\ 1-\frac{x_{ij}}{\sqrt{\sum_{i=1}^{m}x_{ij}^{2}}},\;\;\;\text{for}\;a\;\text{cost}\;\text{criterion} \end{array}\right.$$

(8) $$N7;\;\;\;r_{ij}=\left\{ \begin{array}{c} {\left(\frac{x_{ij}}{{max}_{j}\left(x_{ij}\right)}\right)}^{2},\;\;\;\text{for}\;a\;\text{benefit}\;\text{criterion}\\ {\left(\frac{{min}_{j}\left(x_{ij}\right)}{x_{ij}}\right)}^{3},\;\;\;\text{for}\;a\;\text{cost}\;\text{criterion} \end{array}\right.$$

(9) $$N8;\;\;\;r_{ij}=\left\{ \begin{array}{c} ln\left(x_{ij}\right)/ln\left(\prod_{i=1}^{m}x_{ij}\right),\;\;\;\text{for}\;a\;\text{benefit}\;\text{criterion}\\ \frac{1-ln\left(x_{ij}\right)/ln\left(\prod_{i=1}^{m}x_{ij}\right)}{m-1},\;\;\;\text{for}\;a\;\text{cost}\;\text{criterion} \end{array}\right.$$

(10) $$N9;\;\;\;r_{ij}=\left\{ \begin{array}{c} 1-\frac{{max}_{j}\left(x_{ij}\right)-x_{ij}}{\sum_{i=1}^{m}\left({max}_{j}\left(x_{ij}\right)-x_{ij}\right)},\;\;\;\text{for}\;a\;\text{benefit}\;\text{criterion}\\ 1-\frac{x_{ij}-{min}_{j}\left(x_{ij}\right)}{\sum_{i=1}^{m}\left({x_{ij}-min}_{j}\left(x_{ij}\right)\right)},\;\;\;\text{for}\;a\;\text{cost}\;\text{criterion} \end{array}\right.$$

(11) $$N10;\;\;\;r_{ij}=\left\{ \begin{array}{c} 1-\left|\frac{{max}_{j}\left(x_{ij}\right)-x_{ij}}{{max}_{j}\left(x_{ij}\right)}\right|,\;\;\;\text{for}\;a\;\text{benefit}\;\text{criterion}\\ 1-\left|\frac{{min}_{j}\left(x_{ij}\right)-x_{ij}}{{max}_{j}\left(x_{ij}\right)}\right|,\;\;\;\text{for}\;a\;\text{cost}\;\text{criterion} \end{array}\right.$$

**Table 1:** Survey on past studies according to MCDM techniques and normalization methods.

No.	Source	MCDM technique	N1	N2	N3	N4	N5	N6	N7	N8	N9	N10
1	[26]	Simple Average Weighted (SAW), TOPSIS & Elimination and Choice Translating Reality (ELECTRE)	**×**	✓	**×**	✓	**×**	✓	**×**	**×**	**×**	**×**
2	[27]	TOPSIS	✓	**×**	✓	✓	**×**	✓	**×**	**×**	**×**	**×**
3	[28]	TOPSIS	✓	✓	✓	**×**	**×**	✓	**×**	**×**	**×**	**×**
4	[29]	TOPSIS	✓	✓	✓	**×**	**×**	✓	**×**	**×**	**×**	**×**
5	[30]	TOPSIS	✓	✓	✓	**×**	**×**	✓	**×**	**×**	**×**	**×**
6	[31]	Multi-Criteria Optimization and Compromise Solution (VIKOR)	✓	✓	**×**	✓	**×**	**×**	**×**	**×**	**×**	**×**
7	[32]	TOPSIS	✓	✓	✓	**×**	**×**	✓	**×**	**×**	**×**	**×**
8	[33]	TOPSIS, Preference Ranking Organization Method for Enrichment Evaluation (PROMETHEE) & grey Relational Analysis (GRA)	**×**	✓	**×**	**×**	**×**	✓	✓	**×**	**×**	✓
9	[34]	Mahalanobis Distance-based Ranking Algorithm	✓	✓	✓	**×**	**×**	**×**	**×**	**×**	**×**	**×**
10	[35]	TOPSIS	✓	✓	**×**	**×**	**×**	✓	**×**	✓	**×**	**×**
11	[36]	AHP	✓	✓	✓	**×**	**×**	✓	**×**	✓	**×**	**×**
12	[37]	Weighted Aggregates Sum Product Assessment (WASPAS)	✓	✓	✓	**×**	**×**	✓	**×**	✓	✓	**×**
13	[38]	SAW	✓	✓	✓	**×**	**×**	✓	**×**	✓	**×**	**×**
14	[39]	PROMETHEE	✓	✓	✓	**×**	**×**	✓	**×**	✓	**×**	**×**
15	[40]	Dynamic Multi-Criteria Decision-Making	✓	✓	✓	**×**	**×**	✓	**×**	✓	**×**	**×**
16	[41]	AHP	✓	✓	✓	**×**	**×**	✓	**×**	✓	**×**	**×**
17	[42]	ELECTRE -SAW- TOPSIS	**×**	✓	✓	**×**	**×**	✓	**×**	✓	**×**	**×**
18	[43]	Logic Scoring of Preference	✓	✓	✓	**×**	**×**	✓	**×**	✓	**×**	**×**
19	[44]	Range of Value (ROV)	✓	✓	✓	✓	✓	✓	✓	**×**	✓	**×**
20	[45]	SAW	✓	✓	✓	**×**	**×**	✓	**×**	**×**	**×**	**×**
21	[46]	SAW	✓	✓	✓	**×**	**×**	✓	**×**	✓	**×**	**×**
22	[47]	VIKOR	✓	✓	**×**	✓	**×**	✓	**×**	**×**	✓	**×**

*Note:* (✓) - the method was considered in the comparison; (**×) -** the method was not considered in the comparison; N1 - sum-based method; N2 - maximum-minimum method; N3 - maximum method (version I); N4 - maximum method (version II); N5 - maximum method (version III); N6 - vector method; N7 - non-linear method; N8 - logarithmic method; N9 - enhanced accuracy method; N10 - Jüttler's-Körth's method.

Referring to Table 1, it is apparent that there are at least ten normalization methods that can be tested for their suitability over an MCDM technique. Those methods include the maximum-minimum method [48], the logarithmic method [49], the vector method [50], the enhanced accuracy method [51], and Jüttler's-Körth's method [52]. Table 1 also hints that TOPSIS is the most popular MCDM technique examined for its suitable normalization methods. To be precise, around 40% of the studies in Table 1 focused on TOPSIS, and one of the earliest studies testing TOPSIS with different normalization methods was made by Milani et al. [27]. The test involved N1, N3, N4, and N6, but it only revealed that the use of different normalization methods might lead TOPSIS to deliver varying alternative ranks; the study did not apply any evaluation metrics to compare and decide the most suitable normalization methods.

In contrast to Milani et al. [27], Chakraborty and Yeh [28] compared N1, N2, N3, and N6 using an evaluation metric called Ranking Consistency Index (RCI). With the aid of RCI, they finally recommended TOPSIS's default normalization method, i.e., N6, as the most suitable method, thanks to its ability to produce robust and reliable alternative ranks. A similar study was later conducted by Liao et al. [29], with the same set of normalization methods and evaluation metric, only to reconfirm the recommendation made by Chakraborty and Yeh [28]. Liao et al. [29] also reported that N6 has the advantage of effectively dealing with MCDM problems with various decision matrix sizes, data ranges, and attribute types.

Likewise, the comparison carried out by Celen [30] using RCI once again recommended N6 as the best combination for TOPSIS. Interestingly, his results suggest that N2 or N3 can be a suitable substitute for N6 when applying TOPSIS. The researcher found both N2 and N3 delivered ranks consistent with N6 when he measured the financial performance of 13 deposit banks in Turkish.

In the following year, Vafaei et al. [35] did a slightly different study in which they utilized the average Spearman's rank correlation coefficient as a metric to determine the normalization method that produces the most reliable alternative ranks for TOPSIS. The switch of average Spearman's rank correlation with the common RCI metric is undoubtedly helpful because the computation involving the latter metric becomes exponentially more difficult, especially when a greater number of normalization methods are compared [53].

Lakshmi and Venkatesan [32], on the other hand, compared the suitability of normalization methods over TOPSIS from a rare perspective, the usability perspective. They compared how well methods like N1, N2, N3, and N6 work over the TOPSIS based on two usability metrics, i.e., time complexity and space complexity. They lastly concluded N1 is the best method since it consumes the least time and space. This conclusion goes against the findings of Liao et al. [29], who reported N6 as the most recommendable normalization method for TOPSIS, mainly because Liao et al. [29] performed the comparison based on the result reliability perspective, not usability. The discrepancy in the final recommendations made in these two studies demonstrates that the evaluation metrics employed can influence the selection of suitable normalization methods.

As such, recent attempts have begun using more metrics to evaluate the suitability of various normalization methods over an MCDM technique, e.g. [44]. Usually, a popular voting technique, namely the plurality technique, is used to aggregate the different performance ranks obtained by a normalization method across such metrics to finalize its suitability [46]. To simplify, evaluation metrics for normalization methods can be categorized into three [54]. The first category metrics, like RCI and average Spearman's rank correlation, are used to evaluate the suitability of normalization methods based on their ability to produce reliable alternative ranks [42]. Metrics from the second category, such as the average Pearson correlation coefficient, evaluate suitability based on a method's ability to generate reliable alternative scores [41]. The third category metrics, for example, standard deviation, are used to measure a method's suitability based on its ability to spread distinctive scores across the most and least preferred alternatives [40]. However, none of the existing TOPSIS-related studies tested the suitability of the normalization methods based on the said three perspectives, thus leading to a biased conclusion where a less suitable normalization method is recommended for the TOPSIS application. In conclusion, our survey of past literature uncovers the following limitations:

(a) **Comparison based on a limited range of normalization methods** - To date, no single study has simultaneously compared the suitability of all the ten normalization methods identified in Table 1 for an MCDM technique, including TOPSIS. The maximum number of normalization methods considered in previous TOPSIS-based studies was four, e.g. [35]. Therefore, the conclusiveness of the prior recommendations can be argued. For instance, Chatterjee and Chakraborty [33]  considered N2, N6, N7, and N10 to examine the suitability of these four methods for a few MCDM techniques, including TOPSIS. Their investigation recommended N6 and N10 as the most and least preferred methods for TOPSIS, respectively. However, they could have reached a different recommendation if a more exhaustive array of normalization methods had been considered. They indeed provided numerical proof that TOPSIS is an MCDM technique that is very sensitive to different normalization methods, indicating the immediate need for conclusive recommendations on the suitable normalization methods for TOPSIS.
(b) **Noncomprehensive evaluation procedure** - The literature review discloses that the suitability of a normalization method over an MCDM technique must be evaluated based on three essential perspectives. Those perspectives are the method's ability to produce reliable alternative ranks, generate reliable alternative scores, and spread distinctive scores across the most and least preferred alternatives. Also, the plurality technique is generally utilized to synthesize different performance ranks obtained by a normalization method across the evaluation metrics to finalize its overall suitability. Unfortunately, the available studies on TOPSIS only evaluated suitability based on the alternative ranks’ or scores’ reliability; evaluation based on the spread of alternative scores was absent, e.g. [35]. On the other hand, the plurality technique is known to be non-effective in capturing the relative ranks across various metrics [55], thus may lead to biased suitability results. This limitation signals the urgency for an alternate, comprehensive procedure to evaluate and select suitable normalization methods for TOPSIS.
(c) **Conflicting recommendations** - Literature reports mixed and sometimes conflicting recommendations on suitable normalization methods for TOPSIS. For example,  Celen [30] proposed N2 as one of the normalization methods for TOPSIS, but on the contrary, Vafaei et al. [35] claimed N2 is an absolutely unsuitable choice. The crux of this issue was the lack of comprehensiveness in the context of the range of normalization methods compared and/or the procedure used to evaluate suitability. These mixed, uncertain recommendations in the literature leave the users in a great puzzle as to which is the safer choice of normalization methods for TOPSIS. As such, the room for offering concrete recommendations regarding suitable normalization methods is still available, where such recommendations will lead to safer and more confident decision-making.

Owing to the above limitations, this study aimed to use an inclusive evaluation procedure to make more conclusive recommendations on the suitable BCC-based normalization methods for TOPSIS (out of ten methods traced from previous literature). The contribution of this study is threefold:

(a) **Literature contribution** - This study is the first inclusive endeavor in the current literature to test TOPSIS with all the ten BCC-based normalization methods listed in Table 1.
(b) **Methodological contribution** - The study employed an alternate, comprehensive procedure to evaluate each normalization method's suitability. The procedure was developed based on three evaluation metrics, namely the average Spearman's rank correlation, average Pearson correlation, and standard deviation metrics, combined with the Borda count technique. This interesting combination allows us to select the normalization methods with decent performance across all three metrics to be selected as the ideal ones. Table 2 clarifies the differences between our proposed evaluation procedure and other procedures reported in similar TOPSIS studies based on four characteristics. In short, our proposed procedure has the following four merits:

**Table 2:** Proposed evaluation procedure vs similar procedures in the literature.

Evaluation procedure	Characteristic			
	Evaluation of a method's ability to produce reliable alternative ranks	Evaluation of a method's ability to produce reliable alternative scores	Evaluation of a method's ability to spread distinctive alternative scores	Capture relative ranks across different evaluation metrics in finalizing each method's suitability
[27]	No (Disadvantage)	No (Disadvantage)	No (Disadvantage)	No (Disadvantage)
[28]	Yes (Advantage)	No (Disadvantage)	No (Disadvantage)	No (Disadvantage)
[29]	Yes (Advantage)	No (Disadvantage)	No (Disadvantage)	No (Disadvantage)
[30]	Yes (Advantage)	No (Disadvantage)	No (Disadvantage)	No (Disadvantage)
[35]	No (Disadvantage)	Yes (Advantage)	No (Disadvantage)	No (Disadvantage)
Proposed evaluation procedure	Yes (Advantage)	Yes (Advantage)	Yes (Advantage)	Yes (Advantage)
(c) It uses the average Spearman's rank correlation metric to evaluate each normalization method's ability to produce reliable alternative ranks,
(d) It uses the average Pearson correlation metric to compare each normalization method's ability to produce reliable alternative scores,
(e) It uses the standard deviation metric to test each normalization method's ability to distribute distinctive scores across the most and least preferred alternatives, and
(f) It mathematically captures the relative ranks attained by a normalization method across different evaluation metrics in determining the method's overall suitability.
(g) **Practical contribution** - This study sends a clear message to the users that they should consider combining N4 and N6 into the TOPSIS application for a more reliable result. In other words, they are recommended to aggregate the ranks resulting from both methods to facilitate safer decision-making. In addition, definitive recommendations made through this study could convince users that they are employing the optimal normalization methods, allowing TOPSIS-based decisions to be executed with a better sense of confidence.

## Methodology

We developed a real decision matrix involving 30 alternatives, i.e. smartphone models in the current marketplace, to serve as the primary input for our comparative study (see Table 3). The decision matrix compiles the data of 30 alternatives, $A_{1},\;A_{2}$ ,*…,*$A_{30}$  based on five smartphone criteria suggested by [56]. Those criteria are the price measured in Malaysian Ringgit ($C_{1}$), the screen size measured in inches ($C_{2}$), the pixel density measured in pixels per centimetre ($C_{3}$), the thickness measured in millimetres ($C_{4}$), and the mass measured in grams ($C_{5}$). The data were gleaned from various popular gadget websites, e.g. www.stuff.tv, https://www.techadvisor.com, and https://technave.com/gadget. In other words, the size of the decision matrix is sufficiently large to yield reliable results, and it has a mixture of both the benefit and cost criteria measured with different units, making it suitable for this study. Note that $C_{1}$, $C_{4}$, and $C_{5}$ are cost criteria, and the remaining are benefit criteria.

**Table 3:** Decision matrix of 30 smartphone models.

Model	Price in Malaysia Ringgit ($C_{1}$)	Screen size in inches ($C_{2})$	Pixel density in pixels per centimetre ($C_{3}$)	Thickness in millimetres ($C_{4}$)	Mass in grams ($C_{5}$)
A1	4709	6.8	200	8.9	234
A2	4999	6.1	48	7.85	206
A3	1328	6.7	50	8.9	212
A4	3299	6.7	50	8.5	205
A5	3632	6.7	50	8.5	218
A6	1870	6.74	50	8.9	199
A7	3199	5.4	12	7.65	141
A8	1969	6.1	12	8.9	178
A9	5199	6.5	12	8.2	185
A10	3089	6.7	50	7.3	183
A11	4759	6.2	12	6.3	263
A12	1699	5.9	50	9.1	169
A13	2699	6.78	50	10.3	239
A14	5999	7.9	54	6.1	261
A15	4299	6.81	50	8.8	219
A16	3499	6.73	54	7.8	191
A17	3863	6.8	50	16	191
A18	3499	6.73	50	8.4	210
A19	3220	6.36	50	8	185
A20	3099	6.8	50	8.9	228
A21	3359	6.78	12	8.5	196
A22	4272	6.78	50.3	9.3	214.9
A23	4099	6.74	50	8.5	205
A24	7999	7.8	50	11.1	255
A25	2699	6.67	108	9.5	220
A26	3399	6.81	50	8.4	234
A27	2999	6.92	64	9.9	259
A28	3589	6.8	108	8.9	228
A29	1499	6.67	200	8.9	208.4
A30	4319	6.78	50	9.1	215

Source: www.stuff.tv, https://www.techadvisor.com, and https://technave.com/gadget.

All in all, the study's investigation based on the developed decision matrix can be summarized into three major stages. The stages and steps involved in each stage are outlined in the following sub-sections.

**Stage 1 - Application of TOPSIS with the combination of different normalization methods**

Stage 1 aimed at determining the scores and ranks of the alternatives available in the decision matrix by employing TOPSIS with the combination of ten normalization methods listed in Table 1. Following are the exact steps involved in Stage 1:

(a) **Step 1**- We first normalized the data in the decision matrix using the N1 method to convert them into a common scale using (2). At the end of Step 1, a normalized matrix as expressed by (12) was derived.
  (12) $$\left[r_{ij}\right]=\left[\begin{array}{cccc} r_{11} & r_{12} & \cdots & r_{1n}\\ r_{21} & r_{22} & \cdots & r_{2n}\\ \vdots & \vdots & \vdots & \vdots \\ r_{m1} & r_{m2} & \cdots & r_{mn} \end{array}\right]$$

where $r_{ij}$ is the normalized data of alternative i over criterion j, where i=1,2,…,m and j=1,2,…,n.

(b) **Step 2** - We assigned the weight for each criterion, $w_{j}$ and developed the weighted normalized matrix, $\left[V_{ij}\right]$ as expressed in (13). Note that for this study, we used the criteria weights estimated by Krishnan et al. [56], where these weights were estimated via the combination of various objective weighting techniques. Table 4 shows the weight of each criterion.
  (13) $$\left[V_{ij}\right]=\left[\begin{array}{cccc} V_{11} & V_{12} & \cdots & V_{1n}\\ V_{21} & V_{22} & \cdots & V_{2n}\\ \vdots & \vdots & \vdots & \vdots \\ V_{m1} & V_{m2} & \cdots & V_{mn} \end{array}\right],$$

**Table 4:** The weight of each criterion.

Criterion	$C_{1}$	$C_{2}$	$C_{3}$	$C_{4}$	$C_{5}$
Weight	0.2021	0.1956	0.2031	0.1874	0.2118

Source: [56].

where $V_{ij}=w_{j}\times r_{ij}$.

(c) **Step 3** - We determined the positive-ideal, $A^{*}$ and negative-ideal, $A^{-}$  solutions based on the weighted normalized matrix, using Eqs. (14) and (15).
  (14) $$A^{*}=\left\{V_{1}^{*},V_{2}^{*},\ldots ,V_{n}^{*}\right\}$$
  (15) $$A^{-}=\left\{V_{1}^{-},V_{2}^{-},\ldots ,V_{n}^{-}\right\}$$

where $V_{j}^{*}=maxV_{ij}$; $V_{j}^{-}=minV_{ij}$;i=1,2,…,m; j=1,2,…,n.

(d) **Step 4** - We calculated the separation measure for alternatives using n-dimensional Euclidean distance. The separation/distance of each alternative from the positive-ideal solution, $S_{i}^{*}$, can be obtained using Eq. (16). Similarly, the separation of each alternative from the negative-ideal solution, $S_{i}^{-}$ can be computed based on Eq. (17).
  (16) $$S_{i}^{*}=\sqrt{\sum_{j=1}^{n}{\left(V_{ij}-V_{j}^{*}\right)}^{2}};i=1,2,\ldots ,m$$
  (17) $$S_{i}^{-}=\sqrt{\sum_{j=1}^{n}{\left(V_{ij}-V_{j}^{-}\right)}^{2}};i=1,2,\ldots ,m$$
(e) **Step 5** - We determined the final alternative score for each alternative, $P_{i}$, using Eq. (18), with i=1,2,…,m. The alternatives were then ranked based on $P_{i}$, where higher $P_{i}$ indicates better preference over alternative i.
  (18) $$P_{i}=\frac{S_{i}^{-}}{S_{i}^{*}+S_{i}^{-}};i=1,2,\ldots ,m$$
(f) **Step 6** - Steps 1–5 were repeated based on the other nine normalization methods by properly applying equations (3) - (11). Note that we performed all the calculations with the aid of Microsoft Excel.

**Stage 2 - Evaluation of normalization methods based on three metrics**

In Stage 2, the results obtained from all the different normalization methods were further evaluated based on the three metrics, namely, average Spearman's rank correlation metric, average Pearson correlation metric, and standard deviation metric, to ensure a comprehensive suitability check. Each metric's role and computational steps are available in the following explanation.

(a) **Step 1** - The alternative ranks resulting from all ten methods were analyzed using Spearman's rank correlation test. The test is notably applied to determine the degree of consistency between two different sets of ranks [57]. In other words, this test was carried out to understand each method's ability to provide reliable alternative ranks. The Spearman's correlation coefficient between any two ranks was computed using Eq. (19) [58,59].
  (19) $$k_{s}=1-\frac{6\sum d_{i}^{2}}{m\left(m^{2}-1\right)},$$

where $k_{s}\;$= Spearman's rank correlation coefficient between the alternative ranks resulting from normalization methods, x and y;   $d_{i}=\;$  rank difference for an alternative i based on the normalization methods, x and y; m= total alternatives; i=1,2,…,m. The average $k_{s}$ corresponding to each normalization was then computed, where higher $k_{s}$  implies the method's better ability to produce reliable alternative ranks [60]. 

(b) **Step 2** - The Pearson correlation coefficient is a statistical measure that is widely used to study the degree of consistency between two interval or ratio-based variables [61]. As such, this study performed the Pearson correlation test to measure each normalization method's ability to generate reliable alternative scores. The test indeed has been employed by various scholars for a similar purpose. For example, Kabassi et al. [62] analyzed the Pearson correlations between the scores of four museum websites estimated by different MCDM methods, including fuzzy VIKOR and TOPSIS,  in search of the most reliable method.

The Pearson correlation coefficient between every two sets of alternative scores was calculated using Eq. (20) [63].

(20) $$r_{s}=\frac{\sum \left(P_{ix}-{\bar{P}}_{x}\right)\left(P_{iy}-{\bar{P}}_{y}\right)}{\sqrt{\sum {\left(P_{ix}-{\bar{P}}_{x}\right)}^{2}\sum {\left(P_{iy}-{\bar{P}}_{y}\right)}^{2}}}$$

where $r_{s}\;$= Pearson correlation coefficient of alternative scores identified using normalization method, x and y; $P_{ix}$ = score of an alternative i identified using the normalization method, x; $P_{iy}$ = score of an alternative i identified using the normalization method, y; $\bar{P_{x}}$ = average alternative score of normalization method, k; $\bar{P_{y}}$ = average alternative score of normalization method, y; i=1,2,..,m.

Note that a $r_{s}$ value ranges between −1 to 1. A value closer to 1 indicates higher consistency/similarity, and vice versa if it is closer to −1 [64]. The calculated Rs values were then averaged out according to each normalization method, where higher $r_{s}$  implies the method's better ability to estimate reliable alternative scores [65].

(c) **Step 3** - The standard deviation of the alternative scores resulting from each normalization method was computed using Eq. (21), where a higher standard deviation indicates the method's better ability to distribute distinctive scores across the least to most preferred alternatives.
  (21) $${SD}_{x}=\sqrt{\frac{\sum_{i=1}^{m}{\left(P_{ix}-{\bar{P}}_{x}\right)}^{2}}{m-1}}$$

where $SD_{x}$= standard deviation of a normalization method, x;  $P_{ix}$ = score of an alternative, i by a normalization method, x; $\bar{P_{x}}$ = average alternative scores of normalization method, x; m = total alternatives; i=1,2,..,m.

**Stage 3- Finalization of suitable normalization methods**

Since the normalization methods showcased different performance ranks across all three evaluation metrics, it was difficult to conclude which methods suit TOPSIS. Thus, the analysis was furthered by aggregating these ranks by applying the Borda technique. Although some earlier studies used the plurality technique to aggregate such ranks, this study decided to opt for the Borda technique. The decision was made in light of the technique's ability to control information loss by mathematically capturing the entire relative ranks across different evaluation metrics [66,67]. Also, previous findings hint that the technique can be wise enough to ensure only the normalization method that has acceptable decent performance across all the metrics is chosen to be the best [68].

Following were the steps adhered to determine the final suitability rank of each normalization method using the Borda technique [69,70]:

(a) **Step 1**- Points were assigned to the normalization methods according to their rank over the first metric, i.e., the average Spearman's rank correlation metric. The method ranked first was assigned with (totalranks−1) points, (totalranks−2) points for the method ranked second, and so.
(b) **Step 2**- Step 1 was repeated based on the other two metrics, i.e. the average Pearson correlation metric and standard deviation metric.
(c) **Step 3**- The aggregated Borda score of each normalization was then obtained by summing up the points.
(d) **Step 4**: The normalization methods were re-ranked ascendingly based on the aggregated Borda scores, where each score implies the method's overall suitability over TOPSIS. A higher Borda score, of course, indicates that the method is more suitable for TOPSIS.

**Fig. 1:**
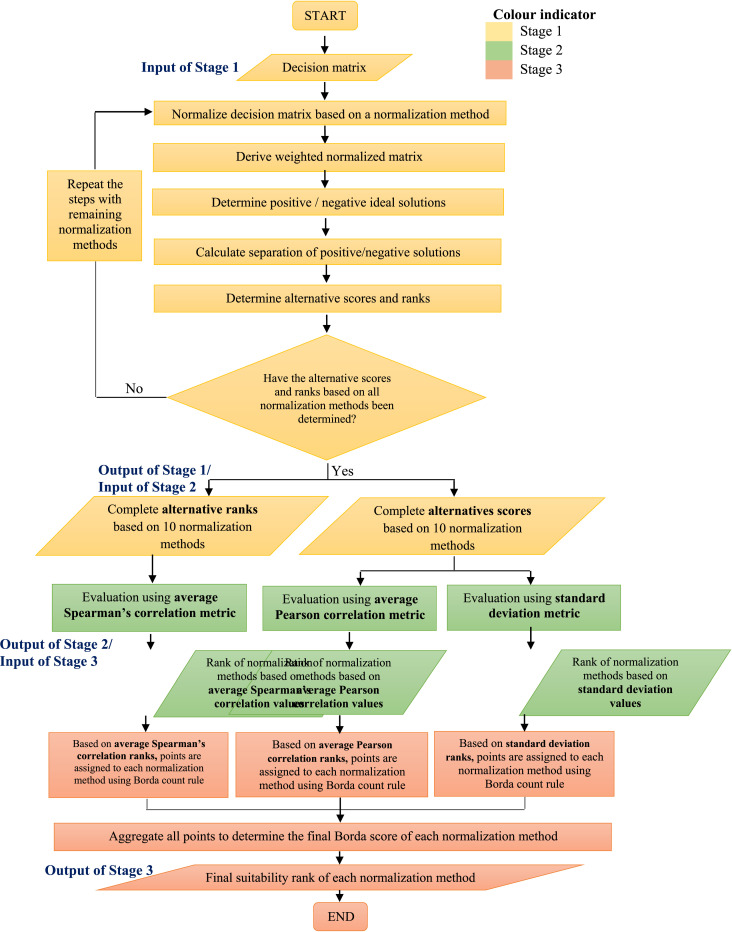
flowchart for Stages 1, 2, and 3.

The flowchart and pseudocode illustrating the inputs, steps and outputs of all three stages are presented via Fig. 1 and Table 5 to allow easy understanding and quick conversion into a proper programming language for future use.

**Table 5:** Pseudocode for Stages 1, 2 and 3.

**Algorithm 1** Pseudocode for Stage 1- Application of TOPSIS with the combination of different normalization methods
1: matrix ← generate(alt, crit) //Generate decision matrix 2: *N* ← 10 // *N* is normalization methods 3:**for***i* = 1 to *N***do** //Step 1: Normalize decision matrix 4:norm_score ← norm_matrix(matrix) //Step 2: Derive weighted normalized matrix 5: weight_score ← weight_matrix(norm_score, crit_weight) //Step 3: Determine positive / negative ideal solutions 6: **for** num_of_weight_score[n] **do** 7: positive ← max(weight_score) 8: negative ← min(weight score) //Step 4: Calculate separation of positive/negative solutions 9: calculate_separation(positive, negative) //Step 5: Determine alternative scores and ranks 10: **for** num_of_alt[n] **do** 11: alt_score ← (positive/sum(positive+negative)) 12: alt_rank(alt_score) 13: **end for** 14: **end for** //Step 6: Repeat Step 1–5 for other 9 methods in loop 15: **end for**
**Algorithm 2** Pseudocode for Stage 2-Evaluation of normalization methods based on three metrics
1: *N* ← 10 // *N* is normalization methods 2: **for***i* = 1 to *N***do** //Step 1: Spearman's rank correlation test 3: spm_rank ← avg[*calculate*_*correlations*(*alt*_*rank*=*i, alt*_*rank*=*i*)] //Step 2: Pearson correlation coefficient 4: pearson_rank ← avg[calculate_correlations(alt_rank=*i*, alt_rank=*i*)] //Step 3: Standard deviation of alternative scores 5: stdv_rank ← calculate_stdv(alt_score) 6: **end for**
**Algorithm 3** Pseudocode for Stage 3- Finalization of suitable normalization methods
1: *N* ← 10 // *N* is normalization methods 2: **for***i* = 1 to *N***do** 3: total_rank ← count(alt_rank) //Step 1: Assign points to N based on their rank Spearman's rank 4: spm_point ← assign_point(total_rank - spm_rank) //Step 2: Assign points to N based on Pearson rank 5: pearson_point ← assign_point(total_rank - pearson_rank) //Step 3: Assign points to N based on standard deviation rank 6: stdv_point ← assign_point(total_rank - stdv_rank) //Step 4: Aggregate Borda points 7: borda_score ← sum(pearson_point, spm_point, stdv_point) //Step 5: *Re*-ranking based on Borda scores 8: ascend_rank(borda_score) 9: **end for**

## Results

Table 6, Table 7, Table 8, Table 9, Table 10, Table 11, Table 12, Table 13, Table 14, Table 15 present the normalized matrices resulting from all ten normalization methods. It is evident that each method has generated a distinct normalized data pattern, as each method transformed data based on various statistical principles or measures, such as the maximum, minimum, sum, and sum of square values. Following is the summary of the patterns we observe in the normalized data structure:

(a) The normalized data in Table 6, Table 7, Table 8, Table 9, Table 10, Table 11, Table 12, Table 13, Table 14, Table 15 are distributed within the interval [0,1].
(b) N1 ensures the sum of each criterion's normalized data equals 1.
(c) N2 transforms the least and the most preferred value of a benefit or cost criteria to 0 and 1, respectively.
(d) Unlike N2, N3 only transforms the most preferred value of a benefit criterion to 1 and the least preferred value of a cost criterion to 0.
(e) N4, N7, N9, and N10, on the other hand, only transform the most preferred value of a benefit or cost criterion to 1.
(f) N5 transforms the most preferred value of a benefit criterion to 1.
(g) N6 neither transforms the most preferred value of a criterion to 1 nor the least preferred value to 0.
(h) Similar to N6, N8 neither transforms the most preferred value of a criterion to 1 nor the least preferred value to 0. In addition, we can also spot a weak distinction in the data normalized by N8, especially for the cost criteria.

**Table 6:** Normalized matrix based on N1.

Alternative/criterion	$C_{1}$	$C_{2}$	$C_{3}$	$C_{4}$	$C_{5}$
A1	0.0218	0.0340	0.1145	0.0322	0.0296
A2	0.0206	0.0305	0.0275	0.0366	0.0336
A3	0.0774	0.0335	0.0286	0.0322	0.0327
A4	0.0312	0.0335	0.0286	0.0338	0.0338
A5	0.0283	0.0335	0.0286	0.0338	0.0318
A6	0.0550	0.0337	0.0286	0.0322	0.0348
A7	0.0321	0.0270	0.0069	0.0375	0.0491
A8	0.0522	0.0305	0.0069	0.0322	0.0389
A9	0.0198	0.0325	0.0069	0.0350	0.0375
A10	0.0333	0.0335	0.0286	0.0393	0.0379
A11	0.0216	0.0310	0.0069	0.0455	0.0263
A12	0.0605	0.0295	0.0286	0.0315	0.0410
A13	0.0381	0.0339	0.0286	0.0279	0.0290
A14	0.0171	0.0395	0.0309	0.0470	0.0265
A15	0.0239	0.0340	0.0286	0.0326	0.0316
A16	0.0294	0.0336	0.0309	0.0368	0.0363
A17	0.0266	0.0340	0.0286	0.0179	0.0363
A18	0.0294	0.0336	0.0286	0.0342	0.0330
A19	0.0319	0.0318	0.0286	0.0359	0.0375
A20	0.0332	0.0340	0.0286	0.0322	0.0304
A21	0.0306	0.0339	0.0069	0.0338	0.0354
A22	0.0241	0.0339	0.0288	0.0309	0.0322
A23	0.0251	0.0337	0.0286	0.0338	0.0338
A24	0.0129	0.0390	0.0286	0.0259	0.0272
A25	0.0381	0.0333	0.0618	0.0302	0.0315
A26	0.0303	0.0340	0.0286	0.0342	0.0296
A27	0.0343	0.0346	0.0366	0.0290	0.0268
A28	0.0287	0.0340	0.0618	0.0322	0.0304
A29	0.0686	0.0333	0.1145	0.0322	0.0333
A30	0.0238	0.0339	0.0286	0.0315	0.0322

**Table 7:** Normalized matrix based on N2.

Model/criterion	$C_{1}$	$C_{2}$	$C_{3}$	$C_{4}$	$C_{5}$
A1	0.4932	0.5600	1.0000	0.7172	0.2377
A2	0.4497	0.2800	0.1915	0.8232	0.4672
A3	1.0000	0.5200	0.2021	0.7172	0.4180
A4	0.7045	0.5200	0.2021	0.7576	0.4754
A5	0.6546	0.5200	0.2021	0.7576	0.3689
A6	0.9188	0.5360	0.2021	0.7172	0.5246
A7	0.7195	0.0000	0.0000	0.8434	1.0000
A8	0.9039	0.2800	0.0000	0.7172	0.6967
A9	0.4197	0.4400	0.0000	0.7879	0.6393
A10	0.7360	0.5200	0.2021	0.8788	0.6557
A11	0.4857	0.3200	0.0000	0.9798	0.0000
A12	0.9444	0.2000	0.2021	0.6970	0.7705
A13	0.7945	0.5520	0.2021	0.5758	0.1967
A14	0.2998	1.0000	0.2234	1.0000	0.0164
A15	0.5546	0.5640	0.2021	0.7273	0.3607
A16	0.6746	0.5320	0.2234	0.8283	0.5902
A17	0.6200	0.5600	0.2021	0.0000	0.5902
A18	0.6746	0.5320	0.2021	0.7677	0.4344
A19	0.7164	0.3840	0.2021	0.8081	0.6393
A20	0.7345	0.5600	0.2021	0.7172	0.2869
A21	0.6955	0.5520	0.0000	0.7576	0.5492
A22	0.5587	0.5520	0.2037	0.6768	0.3943
A23	0.5846	0.5360	0.2021	0.7576	0.4754
A24	0.0000	0.9600	0.2021	0.4949	0.0656
A25	0.7945	0.5080	0.5106	0.6566	0.3525
A26	0.6896	0.5640	0.2021	0.7677	0.2377
A27	0.7495	0.6080	0.2766	0.6162	0.0328
A28	0.6611	0.5600	0.5106	0.7172	0.2869
A29	0.9744	0.5080	1.0000	0.7172	0.4475
A30	0.5516	0.5520	0.2021	0.6970	0.3934

**Table 8:** Normalized matrix based on N3.

Model/Criterion	$C_{1}$	$C_{2}$	$C_{3}$	$C_{4}$	$C_{5}$
A1	0.4113	0.8608	1.0000	0.4438	0.1103
A2	0.3750	0.7722	0.2400	0.5094	0.2167
A3	0.8340	0.8481	0.2500	0.4438	0.1939
A4	0.5876	0.8481	0.2500	0.4688	0.2205
A5	0.5459	0.8481	0.2500	0.4688	0.1711
A6	0.7662	0.8532	0.2500	0.4438	0.2433
A7	0.6001	0.6835	0.0600	0.5219	0.4639
A8	0.7538	0.7722	0.0600	0.4438	0.3232
A9	0.3500	0.8228	0.0600	0.4875	0.2966
A10	0.6138	0.8481	0.2500	0.5438	0.3042
A11	0.4051	0.7848	0.0600	0.6063	0.0000
A12	0.7876	0.7468	0.2500	0.4313	0.3574
A13	0.6626	0.8582	0.2500	0.3563	0.0913
A14	0.2500	1.0000	0.2700	0.6188	0.0076
A15	0.4626	0.8620	0.2500	0.4500	0.1673
A16	0.5626	0.8519	0.2700	0.5125	0.2738
A17	0.5171	0.8608	0.2500	0.0000	0.2738
A18	0.5626	0.8519	0.2500	0.4750	0.2015
A19	0.5974	0.8051	0.2500	0.5000	0.2966
A20	0.6126	0.8608	0.2500	0.4438	0.1331
A21	0.5801	0.8582	0.0600	0.4688	0.2548
A22	0.4659	0.8582	0.2515	0.4188	0.1829
A23	0.4876	0.8532	0.2500	0.4688	0.2205
A24	0.0000	0.9873	0.2500	0.3063	0.0304
A25	0.6626	0.8443	0.5400	0.4063	0.1635
A26	0.5751	0.8620	0.2500	0.4750	0.1103
A27	0.6251	0.8759	0.3200	0.3813	0.0152
A28	0.5513	0.8608	0.5400	0.4438	0.1331
A29	0.8126	0.8443	1.0000	0.4438	0.2076
A30	0.4601	0.8582	0.2500	0.4313	0.1825

**Table 9:** Normalized matrix based on N4.

Model/Criterion	$C_{1}$	$C_{2}$	$C_{3}$	$C_{4}$	$C_{5}$
A1	0.2820	0.8608	1.0000	0.6854	0.6026
A2	0.2657	0.7722	0.2400	0.7771	0.6845
A3	1.0000	0.8481	0.2500	0.6854	0.6651
A4	0.4025	0.8481	0.2500	0.7176	0.6878
A5	0.3656	0.8481	0.2500	0.7176	0.6468
A6	0.7102	0.8532	0.2500	0.6854	0.7085
A7	0.4151	0.6835	0.0600	0.7974	1.0000
A8	0.6745	0.7722	0.0600	0.6854	0.7921
A9	0.2554	0.8228	0.0600	0.7439	0.7622
A10	0.4299	0.8481	0.2500	0.8356	0.7705
A11	0.2791	0.7848	0.0600	0.9683	0.5361
A12	0.7816	0.7468	0.2500	0.6703	0.8343
A13	0.4920	0.8582	0.2500	0.5922	0.5900
A14	0.2214	1.0000	0.2700	1.0000	0.5402
A15	0.3089	0.8620	0.2500	0.6932	0.6438
A16	0.3795	0.8519	0.2700	0.7821	0.7382
A17	0.3438	0.8608	0.2500	0.3813	0.7382
A18	0.3795	0.8519	0.2500	0.7262	0.6714
A19	0.4124	0.8051	0.2500	0.7625	0.7622
A20	0.4285	0.8608	0.2500	0.6854	0.6184
A21	0.3954	0.8582	0.0600	0.7176	0.7194
A22	0.3109	0.8582	0.2515	0.6559	0.6561
A23	0.3240	0.8532	0.2500	0.7176	0.6878
A24	0.1660	0.9873	0.2500	0.5495	0.5529
A25	0.4920	0.8443	0.5400	0.6421	0.6409
A26	0.3907	0.8620	0.2500	0.7262	0.6026
A27	0.4428	0.8759	0.3200	0.6162	0.5444
A28	0.3700	0.8608	0.5400	0.6854	0.6184
A29	0.8859	0.8443	1.0000	0.6854	0.6766
A30	0.3075	0.8582	0.2500	0.6703	0.6558

**Table 10:** Normalized matrix based on N5.

Model/Criterion	$C_{1}$	$C_{2}$	$C_{3}$	$C_{4}$	$C_{5}$
A1	0.4227	0.8608	1.0000	0.1750	0.3536
A2	0.4589	0.7722	0.2400	0.1094	0.2471
A3	0.0000	0.8481	0.2500	0.1750	0.2700
A4	0.2464	0.8481	0.2500	0.1500	0.2433
A5	0.2880	0.8481	0.2500	0.1500	0.2928
A6	0.0678	0.8532	0.2500	0.1750	0.2205
A7	0.2339	0.6835	0.0600	0.0969	0.0000
A8	0.0801	0.7722	0.0600	0.1750	0.1407
A9	0.4839	0.8228	0.0600	0.1313	0.1673
A10	0.2202	0.8481	0.2500	0.0750	0.1597
A11	0.4289	0.7848	0.0600	0.0125	0.4639
A12	0.0464	0.7468	0.2500	0.1875	0.1065
A13	0.1714	0.8582	0.2500	0.2625	0.3726
A14	0.5839	1.0000	0.2700	0.0000	0.4563
A15	0.3714	0.8620	0.2500	0.1688	0.2966
A16	0.2714	0.8519	0.2700	0.1063	0.1901
A17	0.3169	0.8608	0.2500	0.6188	0.1901
A18	0.2714	0.8519	0.2500	0.1438	0.2624
A19	0.2365	0.8051	0.2500	0.1188	0.1673
A20	0.2214	0.8608	0.2500	0.1750	0.3308
A21	0.2539	0.8582	0.0600	0.1500	0.2091
A22	0.3680	0.8582	0.2515	0.2000	0.2810
A23	0.3464	0.8532	0.2500	0.1500	0.2433
A24	0.8340	0.9873	0.2500	0.3125	0.4335
A25	0.1714	0.8443	0.5400	0.2125	0.3004
A26	0.2589	0.8620	0.2500	0.1438	0.3536
A27	0.2089	0.8759	0.3200	0.2375	0.4487
A28	0.2827	0.8608	0.5400	0.1750	0.3308
A29	0.0214	0.8443	1.0000	0.1750	0.2563
A30	0.3739	0.8582	0.2500	0.1875	0.2814

**Table 11:** Normalized matrix based on N6.

Model/Criterion	$C_{1}$	$C_{2}$	$C_{3}$	$C_{4}$	$C_{5}$
A1	0.7770	0.1856	0.5019	0.8195	0.8000
A2	0.7632	0.1665	0.1204	0.8408	0.8239
A3	0.9371	0.1829	0.1255	0.8195	0.8188
A4	0.8437	0.1829	0.1255	0.8276	0.8247
A5	0.8280	0.1829	0.1255	0.8276	0.8136
A6	0.9114	0.1840	0.1255	0.8195	0.8299
A7	0.8485	0.1474	0.0301	0.8448	0.8795
A8	0.9067	0.1665	0.0301	0.8195	0.8478
A9	0.7538	0.1774	0.0301	0.8337	0.8418
A10	0.8537	0.1829	0.1255	0.8519	0.8436
A11	0.7746	0.1692	0.0301	0.8722	0.7752
A12	0.9195	0.1610	0.1255	0.8154	0.8555
A13	0.8722	0.1851	0.1255	0.7911	0.7957
A14	0.7159	0.2156	0.1355	0.8763	0.7769
A15	0.7964	0.1859	0.1255	0.8215	0.8128
A16	0.8343	0.1837	0.1355	0.8418	0.8367
A17	0.8170	0.1856	0.1255	0.6754	0.8367
A18	0.8343	0.1837	0.1255	0.8296	0.8205
A19	0.8475	0.1736	0.1255	0.8377	0.8418
A20	0.8532	0.1856	0.1255	0.8195	0.8051
A21	0.8409	0.1851	0.0301	0.8276	0.8324
A22	0.7977	0.1851	0.1262	0.8113	0.8163
A23	0.8059	0.1840	0.1255	0.8276	0.8247
A24	0.6211	0.2129	0.1255	0.7748	0.7820
A25	0.8722	0.1821	0.2710	0.8073	0.8119
A26	0.8390	0.1859	0.1255	0.8296	0.8000
A27	0.8580	0.1889	0.1606	0.7992	0.7786
A28	0.8300	0.1856	0.2710	0.8195	0.8051
A29	0.9290	0.1821	0.5019	0.8195	0.8218
A30	0.7954	0.1851	0.1255	0.8154	0.8162

**Table 12:** Normalized matrix based on N7.

Model/Criterion	$C_{1}$	$C_{2}$	$C_{3}$	$C_{4}$	$C_{5}$
A1	0.0224	0.7409	1.0000	0.3220	0.2188
A2	0.0187	0.5962	0.0576	0.4692	0.3207
A3	1.0000	0.7193	0.0625	0.3220	0.2942
A4	0.0652	0.7193	0.0625	0.3696	0.3254
A5	0.0489	0.7193	0.0625	0.3696	0.2706
A6	0.3582	0.7279	0.0625	0.3220	0.3557
A7	0.0715	0.4672	0.0036	0.5070	1.0000
A8	0.3068	0.5962	0.0036	0.3220	0.4970
A9	0.0167	0.6770	0.0036	0.4117	0.4427
A10	0.0795	0.7193	0.0625	0.5835	0.4574
A11	0.0217	0.6159	0.0036	0.9078	0.1541
A12	0.4775	0.5578	0.0625	0.3012	0.5808
A13	0.1191	0.7366	0.0625	0.2077	0.2053
A14	0.0108	1.0000	0.0729	1.0000	0.1577
A15	0.0295	0.7431	0.0625	0.3331	0.2669
A16	0.0547	0.7257	0.0729	0.4783	0.4023
A17	0.0406	0.7409	0.0625	0.0554	0.4023
A18	0.0547	0.7257	0.0625	0.3830	0.3027
A19	0.0701	0.6481	0.0625	0.4433	0.4427
A20	0.0787	0.7409	0.0625	0.3220	0.2365
A21	0.0618	0.7366	0.0036	0.3696	0.3723
A22	0.0300	0.7366	0.0633	0.2822	0.2825
A23	0.0340	0.7279	0.0625	0.3696	0.3254
A24	0.0046	0.9748	0.0625	0.1660	0.1691
A25	0.1191	0.7128	0.2916	0.2647	0.2633
A26	0.0596	0.7431	0.0625	0.3830	0.2188
A27	0.0868	0.7673	0.1024	0.2339	0.1613
A28	0.0507	0.7409	0.2916	0.3220	0.2365
A29	0.6953	0.7128	1.0000	0.3220	0.3097
A30	0.0291	0.7366	0.0625	0.3012	0.2821

**Table 13:** Normalized matrix based on N8.

Model/Criterion	$C_{1}$	$C_{2}$	$C_{3}$	$C_{4}$	$C_{5}$
A1	0.0333	0.0337	0.0461	0.0333	0.0333
A2	0.0333	0.0318	0.0337	0.0334	0.0333
A3	0.0335	0.0334	0.0340	0.0333	0.0333
A4	0.0333	0.0334	0.0340	0.0333	0.0333
A5	0.0333	0.0334	0.0340	0.0333	0.0333
A6	0.0334	0.0335	0.0340	0.0333	0.0333
A7	0.0333	0.0297	0.0216	0.0334	0.0334
A8	0.0334	0.0318	0.0216	0.0333	0.0334
A9	0.0333	0.0329	0.0216	0.0334	0.0334
A10	0.0333	0.0334	0.0340	0.0334	0.0334
A11	0.0333	0.0321	0.0216	0.0335	0.0333
A12	0.0334	0.0312	0.0340	0.0333	0.0334
A13	0.0334	0.0337	0.0340	0.0332	0.0333
A14	0.0333	0.0363	0.0347	0.0335	0.0333
A15	0.0333	0.0337	0.0340	0.0333	0.0333
A16	0.0333	0.0335	0.0347	0.0334	0.0334
A17	0.0333	0.0337	0.0340	0.0330	0.0334
A18	0.0333	0.0335	0.0340	0.0334	0.0333
A19	0.0333	0.0325	0.0340	0.0334	0.0334
A20	0.0333	0.0337	0.0340	0.0333	0.0333
A21	0.0333	0.0337	0.0216	0.0333	0.0333
A22	0.0333	0.0337	0.0341	0.0333	0.0333
A23	0.0333	0.0335	0.0340	0.0333	0.0333
A24	0.0332	0.0361	0.0340	0.0332	0.0333
A25	0.0334	0.0334	0.0407	0.0333	0.0333
A26	0.0333	0.0337	0.0340	0.0334	0.0333
A27	0.0333	0.0340	0.0362	0.0333	0.0333
A28	0.0333	0.0337	0.0407	0.0333	0.0333
A29	0.0334	0.0334	0.0461	0.0333	0.0333
A30	0.0333	0.0337	0.0340	0.0333	0.0333

**Table 14:** Normalized matrix based on N9.

Model/Criterion	$C_{1}$	$C_{2}$	$C_{3}$	$C_{4}$	$C_{5}$
A1	0.9505	0.9701	1.0000	0.9660	0.9562
A2	0.9463	0.9511	0.9643	0.9788	0.9694
A3	1.0000	0.9674	0.9647	0.9660	0.9665
A4	0.9712	0.9674	0.9647	0.9709	0.9698
A5	0.9663	0.9674	0.9647	0.9709	0.9637
A6	0.9921	0.9685	0.9647	0.9660	0.9727
A7	0.9726	0.9321	0.9558	0.9812	1.0000
A8	0.9906	0.9511	0.9558	0.9660	0.9826
A9	0.9433	0.9620	0.9558	0.9745	0.9793
A10	0.9742	0.9674	0.9647	0.9854	0.9802
A11	0.9498	0.9538	0.9558	0.9976	0.9425
A12	0.9946	0.9457	0.9647	0.9636	0.9868
A13	0.9799	0.9696	0.9647	0.9490	0.9538
A14	0.9316	1.0000	0.9657	1.0000	0.9435
A15	0.9565	0.9704	0.9647	0.9672	0.9632
A16	0.9682	0.9682	0.9657	0.9794	0.9764
A17	0.9629	0.9701	0.9647	0.8799	0.9764
A18	0.9682	0.9682	0.9647	0.9721	0.9675
A19	0.9723	0.9582	0.9647	0.9769	0.9793
A20	0.9741	0.9701	0.9647	0.9660	0.9590
A21	0.9703	0.9696	0.9558	0.9709	0.9741
A22	0.9569	0.9696	0.9648	0.9612	0.9652
A23	0.9594	0.9685	0.9647	0.9709	0.9698
A24	0.9024	0.9973	0.9647	0.9393	0.9463
A25	0.9799	0.9666	0.9784	0.9587	0.9628
A26	0.9697	0.9704	0.9647	0.9721	0.9562
A27	0.9755	0.9734	0.9680	0.9539	0.9444
A28	0.9669	0.9701	0.9784	0.9660	0.9590
A29	0.9975	0.9666	1.0000	0.9660	0.9682
A30	0.9562	0.9696	0.9647	0.9636	0.9651

**Table 15:** Normalized matrix based on N10.

Model/Criterion	$C_{1}$	$C_{2}$	$C_{3}$	$C_{4}$	$C_{5}$
A1	0.5773	0.8608	1.0000	0.8250	0.6464
A2	0.5411	0.7722	0.2400	0.8906	0.7529
A3	1.0000	0.8481	0.2500	0.8250	0.7300
A4	0.7536	0.8481	0.2500	0.8500	0.7567
A5	0.7120	0.8481	0.2500	0.8500	0.7072
A6	0.9322	0.8532	0.2500	0.8250	0.7795
A7	0.7661	0.6835	0.0600	0.9031	1.0000
A8	0.9199	0.7722	0.0600	0.8250	0.8593
A9	0.5161	0.8228	0.0600	0.8688	0.8327
A10	0.7798	0.8481	0.2500	0.9250	0.8403
A11	0.5711	0.7848	0.0600	0.9875	0.5361
A12	0.9536	0.7468	0.2500	0.8125	0.8935
A13	0.8286	0.8582	0.2500	0.7375	0.6274
A14	0.4161	1.0000	0.2700	1.0000	0.5437
A15	0.6286	0.8620	0.2500	0.8313	0.7034
A16	0.7286	0.8519	0.2700	0.8938	0.8099
A17	0.6831	0.8608	0.2500	0.3813	0.8099
A18	0.7286	0.8519	0.2500	0.8563	0.7376
A19	0.7635	0.8051	0.2500	0.8813	0.8327
A20	0.7786	0.8608	0.2500	0.8250	0.6692
A21	0.7461	0.8582	0.0600	0.8500	0.7909
A22	0.6320	0.8582	0.2515	0.8000	0.7190
A23	0.6536	0.8532	0.2500	0.8500	0.7567
A24	0.1660	0.9873	0.2500	0.6875	0.5665
A25	0.8286	0.8443	0.5400	0.7875	0.6996
A26	0.7411	0.8620	0.2500	0.8563	0.6464
A27	0.7911	0.8759	0.3200	0.7625	0.5513
A28	0.7173	0.8608	0.5400	0.8250	0.6692
A29	0.9786	0.8443	1.0000	0.8250	0.7437
A30	0.6261	0.8582	0.2500	0.8125	0.7186

As expected, the difference in normalized data patterns has led to varying TOPSIS results. Table 16, Table 17, Table 18, Table 19, Table 20, Table 21, Table 22, Table 23, Table 24, Table 25 present the TOPSIS calculations and results derived from each normalization method, including the weighted decision matrix, the positive- and negative-ideal values, the separation measure values, and the alternative scores and ranks. We further summarize the different alternative scores and ranks from all normalization methods in Table 26 to enable easy comparison.

**Table 16:** TOPSIS calculations based on N1.

Weight	0.2021	0.1956	0.2031	0.1874	0.2118				
Alternative/Criterion	$C_{1}$	$C_{2}$	$C_{3}$	$C_{4}$	$C_{5}$	$S^{*}$	$S^{-}$	$P_{i}$	Rank
A1	0.0044	0.0066	0.0233	0.0060	0.0063	0.0123	0.0222	0.6423	2
A2	0.0042	0.0060	0.0056	0.0069	0.0071	0.0215	0.0059	0.2157	25
A3	0.0157	0.0065	0.0058	0.0060	0.0069	0.0180	0.0142	0.4397	4
A4	0.0063	0.0065	0.0058	0.0063	0.0072	0.0202	0.0068	0.2512	16
A5	0.0057	0.0065	0.0058	0.0063	0.0067	0.0206	0.0064	0.2373	19
A6	0.0111	0.0066	0.0058	0.0060	0.0074	0.0185	0.0102	0.3553	7
A7	0.0065	0.0053	0.0014	0.0070	0.0104	0.0239	0.0072	0.2318	20
A8	0.0106	0.0060	0.0014	0.0060	0.0082	0.0228	0.0088	0.2794	10
A9	0.0040	0.0064	0.0014	0.0066	0.0079	0.0250	0.0043	0.1478	30
A10	0.0067	0.0065	0.0058	0.0074	0.0080	0.0198	0.0078	0.2813	9
A11	0.0044	0.0061	0.0014	0.0085	0.0056	0.0251	0.0055	0.1802	28
A12	0.0122	0.0058	0.0058	0.0059	0.0087	0.0182	0.0113	0.3840	6
A13	0.0077	0.0066	0.0058	0.0052	0.0061	0.0200	0.0072	0.2634	14
A14	0.0035	0.0077	0.0063	0.0088	0.0056	0.0214	0.0078	0.2659	12
A15	0.0048	0.0067	0.0058	0.0061	0.0067	0.0211	0.0059	0.2199	22
A16	0.0059	0.0066	0.0063	0.0069	0.0077	0.0199	0.0073	0.2692	11
A17	0.0054	0.0066	0.0058	0.0034	0.0077	0.0212	0.0058	0.2149	26
A18	0.0059	0.0066	0.0058	0.0064	0.0070	0.0204	0.0066	0.2443	17
A19	0.0065	0.0062	0.0058	0.0067	0.0079	0.0200	0.0072	0.2648	13
A20	0.0067	0.0066	0.0058	0.0060	0.0064	0.0202	0.0068	0.2516	15
A21	0.0062	0.0066	0.0014	0.0063	0.0075	0.0242	0.0052	0.1774	29
A22	0.0049	0.0066	0.0059	0.0058	0.0068	0.0210	0.0058	0.2176	23
A23	0.0051	0.0066	0.0058	0.0063	0.0072	0.0208	0.0062	0.2298	21
A24	0.0026	0.0076	0.0058	0.0048	0.0058	0.0226	0.0052	0.1875	27
A25	0.0077	0.0065	0.0126	0.0057	0.0067	0.0142	0.0126	0.4692	3
A26	0.0061	0.0067	0.0058	0.0064	0.0063	0.0205	0.0066	0.2437	18
A27	0.0069	0.0068	0.0074	0.0054	0.0057	0.0190	0.0079	0.2927	8
A28	0.0058	0.0066	0.0126	0.0060	0.0064	0.0154	0.0120	0.4389	5
A29	0.0139	0.0065	0.0233	0.0060	0.0070	0.0049	0.0248	0.8361	1
A30	0.0048	0.0066	0.0058	0.0059	0.0068	0.0211	0.0059	0.2174	24
A*	0.0157	0.0077	0.0233	0.0088	0.0104				
A^-^	0.0026	0.0053	0.0014	0.0034	0.0056

**Table 17:** TOPSIS calculations based on N2.

Weight	0.2021	0.1956	0.2031	0.1874	0.2118				
Alternative/Criterion	$C_{1}$	$C_{2}$	$C_{3}$	$C_{4}$	$C_{5}$	$S^{*}$	$S^{-}$	$P_{i}$	Rank
A1	0.0997	0.1095	0.2031	0.1344	0.0503	0.2163	0.2894	0.5723	3
A2	0.0909	0.0548	0.0389	0.1543	0.0990	0.2702	0.2153	0.4435	27
A3	0.2021	0.1017	0.0411	0.1344	0.0885	0.2304	0.2807	0.5492	7
A4	0.1424	0.1017	0.0411	0.1420	0.1007	0.2303	0.2502	0.5207	11
A5	0.1323	0.1017	0.0411	0.1420	0.0781	0.2447	0.2362	0.4912	17
A6	0.1857	0.1048	0.0411	0.1344	0.1111	0.2184	0.2785	0.5604	4
A7	0.1454	0.0000	0.0000	0.1581	0.2118	0.2891	0.3016	0.5106	14
A8	0.1827	0.0548	0.0000	0.1344	0.1476	0.2615	0.2761	0.5135	12
A9	0.0848	0.0861	0.0000	0.1476	0.1354	0.2728	0.2340	0.4617	25
A10	0.1487	0.1017	0.0411	0.1647	0.1389	0.2092	0.2838	0.5757	2
A11	0.0982	0.0626	0.0000	0.1836	0.0000	0.3386	0.2174	0.3910	29
A12	0.1909	0.0391	0.0411	0.1306	0.1632	0.2376	0.2887	0.5485	8
A13	0.1606	0.1080	0.0411	0.1079	0.0417	0.2663	0.2291	0.4625	24
A14	0.0606	0.1956	0.0454	0.1874	0.0035	0.2972	0.2813	0.4863	19
A15	0.1121	0.1103	0.0411	0.1363	0.0764	0.2502	0.2255	0.4740	21
A16	0.1363	0.1041	0.0454	0.1552	0.1250	0.2148	0.2668	0.5540	5
A17	0.1253	0.1095	0.0411	0.0000	0.1250	0.2867	0.2122	0.4252	28
A18	0.1363	0.1041	0.0411	0.1439	0.0920	0.2350	0.2455	0.5109	13
A19	0.1448	0.0751	0.0411	0.1514	0.1354	0.2263	0.2637	0.5382	9
A20	0.1484	0.1095	0.0411	0.1344	0.0608	0.2493	0.2397	0.4902	18
A21	0.1406	0.1080	0.0000	0.1420	0.1163	0.2528	0.2552	0.5023	15
A22	0.1129	0.1080	0.0414	0.1268	0.0835	0.2488	0.2218	0.4712	23
A23	0.1182	0.1048	0.0411	0.1420	0.1007	0.2365	0.2386	0.5022	16
A24	0.0000	0.1878	0.0411	0.0928	0.0139	0.3395	0.2139	0.3865	30
A25	0.1606	0.0994	0.1037	0.1230	0.0747	0.2093	0.2591	0.5531	6
A26	0.1394	0.1103	0.0411	0.1439	0.0503	0.2558	0.2377	0.4817	20
A27	0.1515	0.1189	0.0562	0.1155	0.0069	0.2778	0.2316	0.4546	26
A28	0.1336	0.1095	0.1037	0.1344	0.0608	0.2182	0.2497	0.5337	10
A29	0.1969	0.0994	0.2031	0.1344	0.0948	0.1606	0.3420	0.6805	1
A30	0.1115	0.1080	0.0411	0.1306	0.0833	0.2488	0.2231	0.4728	22
A*	0.2021	0.1956	0.2031	0.1874	0.2118				
A^-^	0.0000	0.0000	0.0000	0.0000	0.0000

**Table 18:** TOPSIS calculations based on N3.

Weight	0.2021	0.1956	0.2031	0.1874	0.2118				
Alternative/Criterion	$C_{1}$	$C_{2}$	$C_{3}$	$C_{4}$	$C_{5}$	$S^{*}$	$S^{-}$	$P_{i}$	Rank
A1	0.0831	0.1684	0.2031	0.0832	0.0234	0.1213	0.2281	0.6527	2
A2	0.0758	0.1510	0.0487	0.0955	0.0459	0.1938	0.1364	0.4130	25
A3	0.1685	0.1659	0.0508	0.0832	0.0411	0.1686	0.1988	0.5411	6
A4	0.1187	0.1659	0.0508	0.0878	0.0467	0.1732	0.1629	0.4846	11
A5	0.1103	0.1659	0.0508	0.0878	0.0362	0.1792	0.1540	0.4623	19
A6	0.1549	0.1669	0.0508	0.0832	0.0515	0.1657	0.1901	0.5342	7
A7	0.1213	0.1337	0.0122	0.0978	0.0982	0.2070	0.1842	0.4709	15
A8	0.1524	0.1510	0.0122	0.0832	0.0685	0.2016	0.1874	0.4817	12
A9	0.0707	0.1609	0.0122	0.0914	0.0628	0.2215	0.1343	0.3774	28
A10	0.1241	0.1659	0.0508	0.1019	0.0644	0.1656	0.1801	0.5211	8
A11	0.0819	0.1535	0.0122	0.1136	0.0000	0.2354	0.1414	0.3753	29
A12	0.1592	0.1461	0.0508	0.0808	0.0757	0.1658	0.1981	0.5444	5
A13	0.1339	0.1679	0.0508	0.0668	0.0193	0.1839	0.1594	0.4644	17
A14	0.0505	0.1956	0.0548	0.1160	0.0016	0.2127	0.1471	0.4089	26
A15	0.0935	0.1686	0.0508	0.0843	0.0354	0.1858	0.1408	0.4311	22
A16	0.1137	0.1666	0.0548	0.0960	0.0580	0.1669	0.1686	0.5025	10
A17	0.1045	0.1684	0.0508	0.0000	0.0580	0.2076	0.1303	0.3855	27
A18	0.1137	0.1666	0.0508	0.0890	0.0427	0.1757	0.1589	0.4749	13
A19	0.1207	0.1575	0.0508	0.0937	0.0628	0.1694	0.1713	0.5029	9
A20	0.1238	0.1684	0.0508	0.0832	0.0282	0.1787	0.1604	0.4730	14
A21	0.1172	0.1679	0.0122	0.0878	0.0540	0.2064	0.1598	0.4364	21
A22	0.0942	0.1679	0.0511	0.0785	0.0387	0.1854	0.1386	0.4278	24
A23	0.0985	0.1669	0.0508	0.0878	0.0467	0.1799	0.1490	0.4530	20
A24	0.0000	0.1931	0.0508	0.0574	0.0064	0.2519	0.0914	0.2662	30
A25	0.1339	0.1651	0.1097	0.0761	0.0346	0.1284	0.1882	0.5944	3
A26	0.1162	0.1686	0.0508	0.0890	0.0234	0.1817	0.1571	0.4638	18
A27	0.1263	0.1713	0.0650	0.0714	0.0032	0.1802	0.1590	0.4688	16
A28	0.1114	0.1684	0.1097	0.0832	0.0282	0.1368	0.1756	0.5621	4
A29	0.1642	0.1651	0.2031	0.0832	0.0440	0.0705	0.2707	0.7934	1
A30	0.0930	0.1679	0.0508	0.0808	0.0387	0.1857	0.1390	0.4282	23
A*	0.1685	0.1956	0.2031	0.1160	0.0982				
A^-^	0.0000	0.1337	0.0122	0.0000	0.0000

**Table 19:** TOPSIS calculations based on N4.

Weight	0.2021	0.1956	0.2031	0.1874	0.2118				
Alternative/Criterion	$C_{1}$	$C_{2}$	$C_{3}$	$C_{4}$	$C_{5}$	$S^{*}$	$S^{-}$	$P_{i}$	Rank
A1	0.0570	0.1684	0.2031	0.1284	0.1276	0.1799	0.2041	0.5315	2
A2	0.0537	0.1510	0.0487	0.1456	0.1450	0.2325	0.0924	0.2843	23
A3	0.2021	0.1659	0.0508	0.1284	0.1409	0.1805	0.1869	0.5086	3
A4	0.0814	0.1659	0.0508	0.1345	0.1457	0.2141	0.0991	0.3164	14
A5	0.0739	0.1659	0.0508	0.1345	0.1370	0.2212	0.0931	0.2963	21
A6	0.1435	0.1669	0.0508	0.1284	0.1501	0.1864	0.1388	0.4268	6
A7	0.0839	0.1337	0.0122	0.1494	0.2118	0.2360	0.1352	0.3642	11
A8	0.1363	0.1510	0.0122	0.1284	0.1678	0.2195	0.1306	0.3730	9
A9	0.0516	0.1609	0.0122	0.1394	0.1614	0.2552	0.0893	0.2593	28
A10	0.0869	0.1659	0.0508	0.1566	0.1632	0.2017	0.1228	0.3785	8
A11	0.0564	0.1535	0.0122	0.1815	0.1136	0.2629	0.1141	0.3026	18
A12	0.1580	0.1461	0.0508	0.1256	0.1767	0.1807	0.1551	0.4618	4
A13	0.0994	0.1679	0.0508	0.1110	0.1250	0.2188	0.0932	0.2987	20
A14	0.0447	0.1956	0.0548	0.1874	0.1144	0.2371	0.1386	0.3690	10
A15	0.0624	0.1686	0.0508	0.1299	0.1364	0.2290	0.0865	0.2741	25
A16	0.0767	0.1666	0.0548	0.1466	0.1564	0.2081	0.1106	0.3471	12
A17	0.0695	0.1684	0.0508	0.0714	0.1564	0.2409	0.0762	0.2404	29
A18	0.0767	0.1666	0.0508	0.1361	0.1422	0.2174	0.0971	0.3089	15
A19	0.0834	0.1575	0.0508	0.1429	0.1614	0.2080	0.1092	0.3443	13
A20	0.0866	0.1684	0.0508	0.1284	0.1310	0.2175	0.0952	0.3044	17
A21	0.0799	0.1679	0.0122	0.1345	0.1524	0.2418	0.0938	0.2795	24
A22	0.0628	0.1679	0.0511	0.1229	0.1390	0.2297	0.0827	0.2647	27
A23	0.0655	0.1669	0.0508	0.1345	0.1457	0.2233	0.0928	0.2936	22
A24	0.0336	0.1931	0.0508	0.1030	0.1171	0.2602	0.0776	0.2298	30
A25	0.0994	0.1651	0.1097	0.1203	0.1357	0.1746	0.1331	0.4326	5
A26	0.0790	0.1686	0.0508	0.1361	0.1276	0.2209	0.0956	0.3021	19
A27	0.0895	0.1713	0.0650	0.1155	0.1153	0.2164	0.0963	0.3080	16
A28	0.0748	0.1684	0.1097	0.1284	0.1310	0.1889	0.1263	0.4007	7
A29	0.1790	0.1651	0.2031	0.1284	0.1433	0.0981	0.2505	0.7185	1
A30	0.0621	0.1679	0.0508	0.1256	0.1389	0.2295	0.0840	0.2678	26
A*	0.2021	0.1956	0.2031	0.1874	0.2118				
A^-^	0.0336	0.1337	0.0122	0.0714	0.1136

**Table 20:** TOPSIS calculations based on N5.

Weight	0.2021	0.1956	0.2031	0.1874	0.2118				
Alternative/Criterion	$C_{1}$	$C_{2}$	$C_{3}$	$C_{4}$	$C_{5}$	$S^{*}$	$S^{-}$	$P_{i}$	Rank
A1	0.0854	0.1684	0.2031	0.0328	0.0749	0.1229	0.2272	0.6489	1
A2	0.0928	0.1510	0.0487	0.0205	0.0523	0.2068	0.1158	0.3588	12
A3	0.0000	0.1659	0.0508	0.0328	0.0572	0.2472	0.0829	0.2511	24
A4	0.0498	0.1659	0.0508	0.0281	0.0515	0.2193	0.0919	0.2954	21
A5	0.0582	0.1659	0.0508	0.0281	0.0620	0.2128	0.1027	0.3255	17
A6	0.0137	0.1669	0.0508	0.0328	0.0467	0.2400	0.0777	0.2446	27
A7	0.0473	0.1337	0.0122	0.0182	0.0000	0.2724	0.0506	0.1568	29
A8	0.0162	0.1510	0.0122	0.0328	0.0298	0.2706	0.0503	0.1566	30
A9	0.0978	0.1609	0.0122	0.0246	0.0354	0.2344	0.1103	0.3200	19
A10	0.0445	0.1659	0.0508	0.0141	0.0338	0.2324	0.0765	0.2476	25
A11	0.0867	0.1535	0.0122	0.0023	0.0982	0.2405	0.1325	0.3553	13
A12	0.0094	0.1461	0.0508	0.0351	0.0225	0.2515	0.0589	0.1898	28
A13	0.0346	0.1679	0.0508	0.0492	0.0789	0.2162	0.1118	0.3409	14
A14	0.1180	0.1956	0.0548	0.0000	0.0966	0.1949	0.1701	0.4660	4
A15	0.0751	0.1686	0.0508	0.0316	0.0628	0.2026	0.1153	0.3627	10
A16	0.0549	0.1666	0.0548	0.0199	0.0403	0.2198	0.0891	0.2883	22
A17	0.0640	0.1684	0.0508	0.1160	0.0403	0.1955	0.1478	0.4306	6
A18	0.0549	0.1666	0.0508	0.0269	0.0556	0.2161	0.0969	0.3096	20
A19	0.0478	0.1575	0.0508	0.0223	0.0354	0.2280	0.0780	0.2550	23
A20	0.0447	0.1684	0.0508	0.0328	0.0701	0.2168	0.1033	0.3228	18
A21	0.0513	0.1679	0.0122	0.0281	0.0443	0.2482	0.0809	0.2460	26
A22	0.0744	0.1679	0.0511	0.0375	0.0595	0.2010	0.1147	0.3633	9
A23	0.0700	0.1669	0.0508	0.0281	0.0515	0.2089	0.1046	0.3336	16
A24	0.1685	0.1931	0.0508	0.0586	0.0918	0.1629	0.2128	0.5664	2
A25	0.0346	0.1651	0.1097	0.0398	0.0636	0.1860	0.1316	0.4145	7
A26	0.0523	0.1686	0.0508	0.0269	0.0749	0.2143	0.1085	0.3362	15
A27	0.0422	0.1713	0.0650	0.0445	0.0950	0.2018	0.1304	0.3925	8
A28	0.0571	0.1684	0.1097	0.0328	0.0701	0.1720	0.1413	0.4509	5
A29	0.0043	0.1651	0.2031	0.0328	0.0543	0.1917	0.2037	0.5151	3
A30	0.0756	0.1679	0.0508	0.0351	0.0596	0.2016	0.1147	0.3626	11
A*	0.1685	0.1956	0.2031	0.1160	0.0982				
A^-^	0.0000	0.1337	0.0122	0.0000	0.0000

**Table 21:** TOPSIS calculations based on N6.

Weight	0.2021	0.1956	0.2031	0.1874	0.2118				
Alternative/Criterion	$C_{1}$	$C_{2}$	$C_{3}$	$C_{4}$	$C_{5}$	$S^{*}$	$S^{-}$	$P_{i}$	Rank
A1	0.1570	0.0363	0.1019	0.1536	0.1694	0.0385	0.1048	0.7316	2
A2	0.1542	0.0326	0.0245	0.1576	0.1745	0.0867	0.0473	0.3533	26
A3	0.1894	0.0358	0.0255	0.1536	0.1734	0.0785	0.0729	0.4815	5
A4	0.1705	0.0358	0.0255	0.1551	0.1747	0.0804	0.0581	0.4194	13
A5	0.1673	0.0358	0.0255	0.1551	0.1723	0.0815	0.0552	0.4038	17
A6	0.1842	0.0360	0.0255	0.1536	0.1758	0.0783	0.0688	0.4676	7
A7	0.1715	0.0288	0.0061	0.1583	0.1863	0.0986	0.0601	0.3786	20
A8	0.1833	0.0326	0.0061	0.1536	0.1796	0.0973	0.0657	0.4029	18
A9	0.1523	0.0347	0.0061	0.1562	0.1783	0.1036	0.0428	0.2923	29
A10	0.1725	0.0358	0.0255	0.1596	0.1787	0.0790	0.0627	0.4425	8
A11	0.1565	0.0331	0.0061	0.1635	0.1642	0.1041	0.0484	0.3173	28
A12	0.1858	0.0315	0.0255	0.1528	0.1812	0.0783	0.0707	0.4745	6
A13	0.1763	0.0362	0.0255	0.1482	0.1685	0.0814	0.0591	0.4207	12
A14	0.1447	0.0422	0.0275	0.1642	0.1645	0.0895	0.0492	0.3547	25
A15	0.1609	0.0364	0.0255	0.1539	0.1721	0.0836	0.0500	0.3741	21
A16	0.1686	0.0359	0.0275	0.1577	0.1772	0.0783	0.0592	0.4306	10
A17	0.1651	0.0363	0.0255	0.1266	0.1772	0.0893	0.0466	0.3429	27
A18	0.1686	0.0359	0.0255	0.1555	0.1738	0.0809	0.0566	0.4117	15
A19	0.1713	0.0340	0.0255	0.1570	0.1783	0.0797	0.0602	0.4300	11
A20	0.1724	0.0363	0.0255	0.1536	0.1705	0.0808	0.0583	0.4192	14
A21	0.1699	0.0362	0.0061	0.1551	0.1763	0.0989	0.0547	0.3560	24
A22	0.1612	0.0362	0.0256	0.1520	0.1729	0.0835	0.0493	0.3712	22
A23	0.1629	0.0360	0.0255	0.1551	0.1747	0.0825	0.0524	0.3884	19
A24	0.1255	0.0416	0.0255	0.1452	0.1656	0.1035	0.0298	0.2236	30
A25	0.1763	0.0356	0.0550	0.1513	0.1720	0.0528	0.0754	0.5882	3
A26	0.1696	0.0364	0.0255	0.1555	0.1694	0.0814	0.0569	0.4112	16
A27	0.1734	0.0369	0.0326	0.1498	0.1649	0.0758	0.0600	0.4416	9
A28	0.1677	0.0363	0.0550	0.1536	0.1705	0.0553	0.0707	0.5610	4
A29	0.1878	0.0356	0.1019	0.1536	0.1741	0.0176	0.1180	0.8705	1
A30	0.1608	0.0362	0.0255	0.1528	0.1729	0.0837	0.0493	0.3708	23
A*	0.1894	0.0422	0.1019	0.1642	0.1863				
A^-^	0.1255	0.0288	0.0061	0.1266	0.1642

**Table 22:** TOPSIS calculations based on N7.

Weight	0.2021	0.1956	0.2031	0.1874	0.2118				
Alternative/Criterion	$C_{1}$	$C_{2}$	$C_{3}$	$C_{4}$	$C_{5}$	$S^{*}$	$S^{-}$	$P_{i}$	Rank
A1	0.0045	0.1449	0.2031	0.0603	0.0463	0.2918	0.2157	0.4250	3
A2	0.0038	0.1166	0.0117	0.0879	0.0679	0.3359	0.0896	0.2106	18
A3	0.2021	0.1407	0.0127	0.0603	0.0623	0.2789	0.2155	0.4359	2
A4	0.0132	0.1407	0.0127	0.0693	0.0689	0.3307	0.0866	0.2076	19
A5	0.0099	0.1407	0.0127	0.0693	0.0573	0.3377	0.0820	0.1954	23
A6	0.0724	0.1424	0.0127	0.0603	0.0753	0.3011	0.1103	0.2681	9
A7	0.0145	0.0914	0.0007	0.0950	0.2118	0.3091	0.1986	0.3912	4
A8	0.0620	0.1166	0.0007	0.0603	0.1053	0.3071	0.1102	0.2640	10
A9	0.0034	0.1324	0.0007	0.0771	0.0938	0.3325	0.0994	0.2302	14
A10	0.0161	0.1407	0.0127	0.1093	0.0969	0.3053	0.1293	0.2976	8
A11	0.0044	0.1205	0.0007	0.1701	0.0326	0.3436	0.1624	0.3209	7
A12	0.0965	0.1091	0.0127	0.0564	0.1230	0.2827	0.1410	0.3328	6
A13	0.0241	0.1441	0.0127	0.0389	0.0435	0.3478	0.0662	0.1600	30
A14	0.0022	0.1956	0.0148	0.1874	0.0334	0.3275	0.2059	0.3860	5
A15	0.0060	0.1453	0.0127	0.0624	0.0565	0.3420	0.0797	0.1891	24
A16	0.0110	0.1420	0.0148	0.0896	0.0852	0.3169	0.1091	0.2561	11
A17	0.0082	0.1449	0.0127	0.0104	0.0852	0.3518	0.0763	0.1783	27
A18	0.0110	0.1420	0.0127	0.0718	0.0641	0.3329	0.0870	0.2071	20
A19	0.0142	0.1268	0.0127	0.0831	0.0938	0.3180	0.1029	0.2445	12
A20	0.0159	0.1449	0.0127	0.0603	0.0501	0.3403	0.0777	0.1858	25
A21	0.0125	0.1441	0.0007	0.0693	0.0789	0.3335	0.0923	0.2167	17
A22	0.0061	0.1441	0.0128	0.0529	0.0598	0.3442	0.0741	0.1772	28
A23	0.0069	0.1424	0.0127	0.0693	0.0689	0.3340	0.0870	0.2065	21
A24	0.0009	0.1907	0.0127	0.0311	0.0358	0.3635	0.1022	0.2194	16
A25	0.0241	0.1394	0.0592	0.0496	0.0558	0.3145	0.0913	0.2250	15
A26	0.0121	0.1453	0.0127	0.0718	0.0463	0.3401	0.0845	0.1990	22
A27	0.0175	0.1501	0.0208	0.0438	0.0342	0.3486	0.0724	0.1720	29
A28	0.0102	0.1449	0.0592	0.0603	0.0501	0.3200	0.0958	0.2304	13
A29	0.1405	0.1394	0.2031	0.0603	0.0656	0.2109	0.2575	0.5498	1
A30	0.0059	0.1441	0.0127	0.0564	0.0597	0.3431	0.0762	0.1817	26
A*	0.2021	0.1956	0.2031	0.1874	0.2118				
A^-^	0.0009	0.0914	0.0007	0.0104	0.0326

**Table 23:** TOPSIS calculations based on N8.

Weight	0.2021	0.1956	0.2031	0.1874	0.2118				
Alternative/Criterion	$C_{1}$	$C_{2}$	$C_{3}$	$C_{4}$	$C_{5}$	$S^{*}$	$S^{-}$	$P_{i}$	Rank
A1	0.0067	0.0066	0.0094	0.0062	0.0071	0.0005	0.0050	0.9066	1
A2	0.0067	0.0062	0.0068	0.0063	0.0071	0.0027	0.0025	0.4818	25
A3	0.0068	0.0065	0.0069	0.0062	0.0071	0.0025	0.0026	0.5112	20
A4	0.0067	0.0065	0.0069	0.0062	0.0071	0.0025	0.0026	0.5112	21
A5	0.0067	0.0065	0.0069	0.0062	0.0071	0.0025	0.0026	0.5111	22
A6	0.0068	0.0066	0.0069	0.0062	0.0071	0.0025	0.0026	0.5122	16
A7	0.0067	0.0058	0.0044	0.0063	0.0071	0.0051	0.0001	0.0159	30
A8	0.0068	0.0062	0.0044	0.0062	0.0071	0.0051	0.0004	0.0777	29
A9	0.0067	0.0064	0.0044	0.0063	0.0071	0.0050	0.0006	0.1133	27
A10	0.0067	0.0065	0.0069	0.0063	0.0071	0.0025	0.0026	0.5112	19
A11	0.0067	0.0063	0.0044	0.0063	0.0070	0.0050	0.0005	0.0876	28
A12	0.0068	0.0061	0.0069	0.0062	0.0071	0.0026	0.0025	0.4897	24
A13	0.0067	0.0066	0.0069	0.0062	0.0071	0.0025	0.0026	0.5131	15
A14	0.0067	0.0071	0.0071	0.0063	0.0071	0.0023	0.0030	0.5615	6
A15	0.0067	0.0066	0.0069	0.0062	0.0071	0.0025	0.0026	0.5139	11
A16	0.0067	0.0066	0.0071	0.0063	0.0071	0.0024	0.0028	0.5375	7
A17	0.0067	0.0066	0.0069	0.0062	0.0071	0.0025	0.0026	0.5134	13
A18	0.0067	0.0066	0.0069	0.0063	0.0071	0.0025	0.0026	0.5119	18
A19	0.0067	0.0064	0.0069	0.0063	0.0071	0.0026	0.0026	0.5023	23
A20	0.0067	0.0066	0.0069	0.0062	0.0071	0.0025	0.0026	0.5136	12
A21	0.0067	0.0066	0.0044	0.0062	0.0071	0.0050	0.0008	0.1358	26
A22	0.0067	0.0066	0.0069	0.0062	0.0071	0.0025	0.0027	0.5151	9
A23	0.0067	0.0066	0.0069	0.0062	0.0071	0.0025	0.0026	0.5121	17
A24	0.0067	0.0071	0.0069	0.0062	0.0071	0.0025	0.0028	0.5351	8
A25	0.0067	0.0065	0.0083	0.0062	0.0071	0.0012	0.0040	0.7617	4
A26	0.0067	0.0066	0.0069	0.0063	0.0071	0.0025	0.0026	0.5139	10
A27	0.0067	0.0067	0.0074	0.0062	0.0071	0.0021	0.0031	0.5985	5
A28	0.0067	0.0066	0.0083	0.0062	0.0071	0.0012	0.0040	0.7667	3
A29	0.0068	0.0065	0.0094	0.0062	0.0071	0.0006	0.0050	0.8960	2
A30	0.0067	0.0066	0.0069	0.0062	0.0071	0.0025	0.0026	0.5131	14
A*	0.0068	0.0071	0.0094	0.0063	0.0071				
A^-^	0.0067	0.0058	0.0044	0.0062	0.0070

**Table 24:** TOPSIS calculations based on N9.

Weight	0.2021	0.1956	0.2031	0.1874	0.2118				
Alternative/Criterion	$C_{1}$	$C_{2}$	$C_{3}$	$C_{4}$	$C_{5}$	$S^{*}$	$S^{-}$	$P_{i}$	Rank
A1	0.1921	0.1898	0.2031	0.1810	0.2025	0.0162	0.0224	0.5805	19
A2	0.1912	0.1860	0.1958	0.1834	0.2053	0.0179	0.0217	0.5483	27
A3	0.2021	0.1892	0.1959	0.1810	0.2047	0.0135	0.0270	0.6661	4
A4	0.1963	0.1892	0.1959	0.1819	0.2054	0.0140	0.0238	0.6298	8
A5	0.1953	0.1892	0.1959	0.1819	0.2041	0.0151	0.0230	0.6041	17
A6	0.2005	0.1894	0.1959	0.1810	0.2060	0.0129	0.0262	0.6700	3
A7	0.1966	0.1823	0.1941	0.1839	0.2118	0.0173	0.0267	0.6060	15
A8	0.2002	0.1860	0.1941	0.1810	0.2081	0.0152	0.0258	0.6297	9
A9	0.1906	0.1882	0.1941	0.1826	0.2074	0.0176	0.0219	0.5543	26
A10	0.1969	0.1892	0.1959	0.1847	0.2076	0.0120	0.0268	0.6905	2
A11	0.1920	0.1866	0.1941	0.1869	0.1996	0.0203	0.0244	0.5456	28
A12	0.2010	0.1850	0.1959	0.1806	0.2090	0.0148	0.0263	0.6395	7
A13	0.1980	0.1896	0.1959	0.1778	0.2020	0.0170	0.0218	0.5618	23
A14	0.1883	0.1956	0.1961	0.1874	0.1998	0.0196	0.0269	0.5787	20
A15	0.1933	0.1898	0.1959	0.1813	0.2040	0.0161	0.0216	0.5724	21
A16	0.1957	0.1894	0.1961	0.1835	0.2068	0.0130	0.0251	0.6593	5
A17	0.1946	0.1898	0.1959	0.1649	0.2068	0.0260	0.0161	0.3832	30
A18	0.1957	0.1894	0.1959	0.1822	0.2049	0.0144	0.0236	0.6219	12
A19	0.1965	0.1874	0.1959	0.1831	0.2074	0.0137	0.0249	0.6453	6
A20	0.1969	0.1898	0.1959	0.1810	0.2031	0.0151	0.0233	0.6061	14
A21	0.1961	0.1896	0.1941	0.1819	0.2063	0.0146	0.0240	0.6228	11
A22	0.1934	0.1896	0.1960	0.1801	0.2044	0.0164	0.0208	0.5592	24
A23	0.1939	0.1894	0.1959	0.1819	0.2054	0.0151	0.0226	0.6001	18
A24	0.1824	0.1951	0.1959	0.1760	0.2004	0.0265	0.0171	0.3920	29
A25	0.1980	0.1891	0.1987	0.1797	0.2039	0.0142	0.0234	0.6234	10
A26	0.1960	0.1898	0.1959	0.1822	0.2025	0.0154	0.0235	0.6046	16
A27	0.1972	0.1904	0.1966	0.1788	0.2000	0.0175	0.0220	0.5563	25
A28	0.1954	0.1898	0.1987	0.1810	0.2031	0.0146	0.0228	0.6090	13
A29	0.2016	0.1891	0.2031	0.1810	0.2051	0.0113	0.0280	0.7119	1
A30	0.1933	0.1896	0.1959	0.1806	0.2044	0.0163	0.0211	0.5639	22
A*	0.2021	0.1956	0.2031	0.1874	0.2118				
A^-^	0.1824	0.1823	0.1941	0.1649	0.1996

**Table 25:** TOPSIS calculations based on N10.

Weight	0.2021	0.1956	0.2031	0.1874	0.2118				
Alternative/Criterion	$C_{1}$	$C_{2}$	$C_{3}$	$C_{4}$	$C_{5}$	$S^{*}$	$S^{-}$	$P_{i}$	Rank
A1	0.1167	0.1684	0.2031	0.1546	0.1369	0.1213	0.2281	0.6527	2
A2	0.1093	0.1510	0.0487	0.1669	0.1595	0.1938	0.1364	0.4130	25
A3	0.2021	0.1659	0.0508	0.1546	0.1546	0.1686	0.1988	0.5411	6
A4	0.1523	0.1659	0.0508	0.1593	0.1603	0.1732	0.1629	0.4846	11
A5	0.1439	0.1659	0.0508	0.1593	0.1498	0.1792	0.1540	0.4623	19
A6	0.1884	0.1669	0.0508	0.1546	0.1651	0.1657	0.1901	0.5342	7
A7	0.1548	0.1337	0.0122	0.1692	0.2118	0.2070	0.1842	0.4709	15
A8	0.1859	0.1510	0.0122	0.1546	0.1820	0.2016	0.1874	0.4817	12
A9	0.1043	0.1609	0.0122	0.1628	0.1764	0.2215	0.1343	0.3774	28
A10	0.1576	0.1659	0.0508	0.1733	0.1780	0.1656	0.1801	0.5211	8
A11	0.1154	0.1535	0.0122	0.1851	0.1136	0.2354	0.1414	0.3753	29
A12	0.1927	0.1461	0.0508	0.1523	0.1893	0.1658	0.1981	0.5444	5
A13	0.1675	0.1679	0.0508	0.1382	0.1329	0.1839	0.1594	0.4644	17
A14	0.0841	0.1956	0.0548	0.1874	0.1152	0.2127	0.1471	0.4089	26
A15	0.1270	0.1686	0.0508	0.1558	0.1490	0.1858	0.1408	0.4311	22
A16	0.1472	0.1666	0.0548	0.1675	0.1715	0.1669	0.1686	0.5025	10
A17	0.1381	0.1684	0.0508	0.0714	0.1715	0.2076	0.1303	0.3855	27
A18	0.1472	0.1666	0.0508	0.1605	0.1562	0.1757	0.1589	0.4749	13
A19	0.1543	0.1575	0.0508	0.1651	0.1764	0.1694	0.1713	0.5029	9
A20	0.1574	0.1684	0.0508	0.1546	0.1417	0.1787	0.1604	0.4730	14
A21	0.1508	0.1679	0.0122	0.1593	0.1675	0.2064	0.1598	0.4364	21
A22	0.1277	0.1679	0.0511	0.1499	0.1523	0.1854	0.1386	0.4278	24
A23	0.1321	0.1669	0.0508	0.1593	0.1603	0.1799	0.1490	0.4530	20
A24	0.0336	0.1931	0.0508	0.1288	0.1200	0.2519	0.0914	0.2662	30
A25	0.1675	0.1651	0.1097	0.1476	0.1482	0.1284	0.1882	0.5944	3
A26	0.1498	0.1686	0.0508	0.1605	0.1369	0.1817	0.1571	0.4638	18
A27	0.1599	0.1713	0.0650	0.1429	0.1168	0.1802	0.1590	0.4688	16
A28	0.1450	0.1684	0.1097	0.1546	0.1417	0.1368	0.1756	0.5621	4
A29	0.1978	0.1651	0.2031	0.1546	0.1575	0.0705	0.2707	0.7934	1
A30	0.1265	0.1679	0.0508	0.1523	0.1522	0.1857	0.1390	0.4282	23
A*	0.2021	0.1956	0.2031	0.1874	0.2118				
A^-^	0.0336	0.1337	0.0122	0.0714	0.1136

**Table 26:** Alternative scores and ranks.

Method/Alternative	N1		N2		N3		N4		N5	
	Score	Rank	Score	Rank	Score	Rank	Score	Rank	Score	Rank
A1	0.6423	2	0.5723	3	0.6527	2	0.5315	2	0.6489	1
A2	0.2157	25	0.4435	27	0.4130	25	0.2843	23	0.3588	12
A3	0.4397	4	0.5492	7	0.5411	6	0.5086	3	0.2511	24
A4	0.2512	16	0.5207	11	0.4846	11	0.3164	14	0.2954	21
A5	0.2373	19	0.4912	17	0.4623	19	0.2963	21	0.3255	17
A6	0.3553	7	0.5604	4	0.5342	7	0.4268	6	0.2446	27
A7	0.2318	20	0.5106	14	0.4709	15	0.3642	11	0.1568	29
A8	0.2794	10	0.5135	12	0.4817	12	0.3730	9	0.1566	30
A9	0.1478	30	0.4617	25	0.3774	28	0.2593	28	0.3200	19
A10	0.2813	9	0.5757	2	0.5211	8	0.3785	8	0.2476	25
A11	0.1802	28	0.3910	29	0.3753	29	0.3026	18	0.3553	13
A12	0.3840	6	0.5485	8	0.5444	5	0.4618	4	0.1898	28
A13	0.2634	14	0.4625	24	0.4644	17	0.2987	20	0.3409	14
A14	0.2659	12	0.4863	19	0.4089	26	0.3690	10	0.4660	4
A15	0.2199	22	0.4740	21	0.4311	22	0.2741	25	0.3627	10
A16	0.2692	11	0.5540	5	0.5025	10	0.3471	12	0.2883	22
A17	0.2149	26	0.4252	28	0.3855	27	0.2404	29	0.4306	6
A18	0.2443	17	0.5109	13	0.4749	13	0.3089	15	0.3096	20
A19	0.2648	13	0.5382	9	0.5029	9	0.3443	13	0.2550	23
A20	0.2516	15	0.4902	18	0.4730	14	0.3044	17	0.3228	18
A21	0.1774	29	0.5023	15	0.4364	21	0.2795	24	0.2460	26
A22	0.2176	23	0.4712	23	0.4278	24	0.2647	27	0.3633	9
A23	0.2298	21	0.5022	16	0.4530	20	0.2936	22	0.3336	16
A24	0.1875	27	0.3865	30	0.2662	30	0.2298	30	0.5664	2
A25	0.4692	3	0.5531	6	0.5944	3	0.4326	5	0.4145	7
A26	0.2437	18	0.4817	20	0.4638	18	0.3021	19	0.3362	15
A27	0.2927	8	0.4546	26	0.4688	16	0.3080	16	0.3925	8
A28	0.4389	5	0.5337	10	0.5621	4	0.4007	7	0.4509	5
A29	0.8361	1	0.6805	1	0.7934	1	0.7185	1	0.5151	3
A30	0.2174	24	0.4728	22	0.4282	23	0.2678	26	0.3626	11
Method/Alternative	N6		N7		N8		N9		N10	
	Score	Rank	Score	Rank	Score	Rank	Score	Rank	Score	Rank

A1	0.7316	2	0.4250	3	0.9066	1	0.5805	19	0.6527	2
A2	0.3533	26	0.2106	18	0.4818	25	0.5483	27	0.4130	25
A3	0.4815	5	0.4359	2	0.5112	20	0.6661	4	0.5411	6
A4	0.4194	13	0.2076	19	0.5112	21	0.6298	8	0.4846	11
A5	0.4038	17	0.1954	23	0.5111	22	0.6041	17	0.4623	19
A6	0.4676	7	0.2681	9	0.5122	16	0.6700	3	0.5342	7
A7	0.3786	20	0.3912	4	0.0159	30	0.6060	15	0.4709	15
A8	0.4029	18	0.2640	10	0.0777	29	0.6297	9	0.4817	12
A9	0.2923	29	0.2302	14	0.1133	27	0.5543	26	0.3774	28
A10	0.4425	8	0.2976	8	0.5112	19	0.6905	2	0.5211	8
A11	0.3173	28	0.3209	7	0.0876	28	0.5456	28	0.3753	29
A12	0.4745	6	0.3328	6	0.4897	24	0.6395	7	0.5444	5
A13	0.4207	12	0.1600	30	0.5131	15	0.5618	23	0.4644	17
A14	0.3547	25	0.3860	5	0.5615	6	0.5787	20	0.4089	26
A15	0.3741	21	0.1891	24	0.5139	11	0.5724	21	0.4311	22
A16	0.4306	10	0.2561	11	0.5375	7	0.6593	5	0.5025	10
A17	0.3429	27	0.1783	27	0.5134	13	0.3832	30	0.3855	27
A18	0.4117	15	0.2071	20	0.5119	18	0.6219	12	0.4749	13
A19	0.4300	11	0.2445	12	0.5023	23	0.6453	6	0.5029	9
A20	0.4192	14	0.1858	25	0.5136	12	0.6061	14	0.4730	14
A21	0.3560	24	0.2167	17	0.1358	26	0.6228	11	0.4364	21
A22	0.3712	22	0.1772	28	0.5151	9	0.5592	24	0.4278	24
A23	0.3884	19	0.2065	21	0.5121	17	0.6001	18	0.4530	20
A24	0.2236	30	0.2194	16	0.5351	8	0.3920	29	0.2662	30
A25	0.5882	3	0.2250	15	0.7617	4	0.6234	10	0.5944	3
A26	0.4112	16	0.1990	22	0.5139	10	0.6046	16	0.4638	18
A27	0.4416	9	0.1720	29	0.5985	5	0.5563	25	0.4688	16
A28	0.5610	4	0.2304	13	0.7667	3	0.6090	13	0.5621	4
A29	0.8705	1	0.5498	1	0.8960	2	0.7119	1	0.7934	1
A30	0.3708	23	0.1817	26	0.5131	14	0.5639	22	0.4282	23

**Fig. 2:**
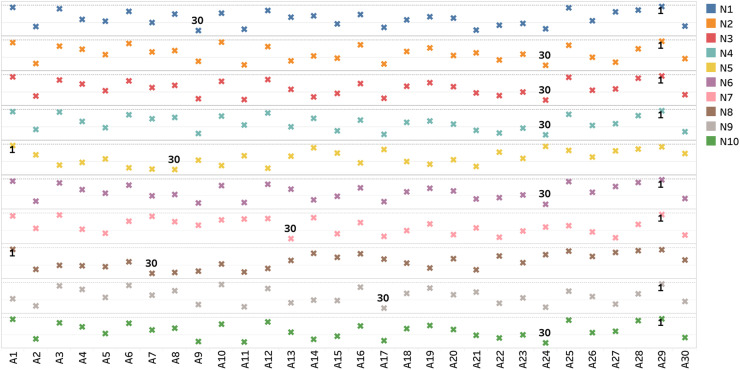
Alternatives in the 1st and 30th ranks according to each normalization method. *Note:* 1 = alternative in the 1st rank; 30 = alternative in the 30th rank.

Nonetheless, based on Fig. 2, which labels the alternatives in the 1st and 30th ranks, it can be noticed that all methods, except N5 and N8, identified A29 as the most preferred alternative. Whereas five methods, i.e., N2, N3, N4, N6, and N10, indicated A24 as the least preferred alternative.

Meanwhile, Table 27 suggests that N1, N2, N3, N4, N6, and N10 are the more suitable methods for TOPSIS in terms of ranking reliability since these six methods scored a higher average Spearman's rank correlation, i.e., which is more than 0.6000. Whereas N5, which recorded the lowest score, i.e., 0.0002, is deemed the least suitable.

**Table 27:** Results of Spearman's rank correlation metric.

	N1	N2	N3	N4	N5	N6	N7	N8	N9	N10
N1	1.0000	0.7780	0.9010	0.9110	0.0060	0.9310	0.4630	0.4480	0.6690	0.9010
N2	0.7780	1.0000	0.9070	0.8430	−0.3310	0.8180	0.6140	0.1460	0.9150	0.9070
N3	0.9010	0.9070	1.0000	0.8820	−0.2110	0.9610	0.4670	0.2540	0.8180	1.0000
N4	0.9110	0.8430	0.8820	1.0000	−0.1840	0.8360	0.7290	0.1810	0.7470	0.8820
N5	0.0060	−0.3310	−0.2110	−0.1840	1.0000	−0.0710	−0.2110	0.7580	−0.5430	−0.2110
N6	0.9310	0.8180	0.9610	0.8360	−0.0710	1.0000	0.3390	0.4000	0.7200	0.9610
N7	0.4630	0.6140	0.4670	0.7290	−0.2110	0.3390	1.0000	−0.1190	0.5220	0.4670
N8	0.4480	0.1460	0.2540	0.1810	0.7580	0.4000	−0.1190	1.0000	−0.0370	0.2540
N9	0.6690	0.9150	0.8180	0.7470	−0.5430	0.7200	0.5220	−0.0370	1.0000	0.8180
N10	0.9010	0.9070	1.0000	0.8820	−0.2110	0.9610	0.4670	0.2540	0.8180	1.0000
Averagee	0.7008	0.6597	0.6979	0.6827	0.0002	0.6895	0.4271	0.3285	0.5629	0.6979
Rank	1	6	2N	5	10	4	8	9	7	2

On the other hand, in the context of reliable alternative scores, based on Table 28, it can be claimed that N6 is the most suitable method for TOPSIS, with an average score of  0.7795. N1, with an average of 0.7766, is considered the second-most suitable method. N5, with the lowest average, is again reported as the poorest combination for TOPSIS.

**Table 28:** Results of Pearson's correlation metric. .

	N1	N2	N3	N4	N5	N6	N7	N8	N9	N10
N1	1.0000	0.7839	0.9159	0.9432	0.4125	0.9664	0.7067	0.6760	0.4454	0.9159
N2	0.7839	1.0000	0.9203	0.8507	−0.0794	0.8334	0.6063	0.4484	0.8230	0.9203
N3	0.9159	0.9203	1.0000	0.9087	0.1109	0.9708	0.6046	0.5677	0.7066	1.0000
N4	0.9432	0.8507	0.9087	1.0000	0.1624	0.8968	0.8466	0.4755	0.6136	0.9087
N5	0.4125	−0.0794	0.1109	0.1624	1.0000	0.3370	0.1390	0.6671	−0.4477	0.1109
N6	0.9664	0.8334	0.9708	0.8968	0.3370	1.0000	0.6017	0.6736	0.5450	0.9708
N7	0.7067	0.6063	0.6046	0.8466	0.1390	0.6017	1.0000	0.1550	0.4200	0.6046
N8	0.6760	0.4484	0.5677	0.4755	0.6671	0.6736	0.1550	1.0000	0.1081	0.5677
N9	0.4454	0.8230	0.7066	0.6136	−0.4477	0.5450	0.4200	0.1081	1.0000	0.7066
N10	0.9159	0.9203	1.0000	0.9087	0.1109	0.9708	0.6046	0.5677	0.7066	1.0000
Average	0.7766	0.7107	0.7705	0.7606	0.2413	0.7795	0.5685	0.5339	0.4921	0.7705
Rank	2	6	3	5	10	1	7	8	9	3

On the contrary, in terms of variation in alternative scores, N8 with the highest standard deviation, i.e., 0.2151, appears as the most suitable method, followed by N1 > N6 > N5 > N4 > N7 > N3 and N10 > N9 > N10 (refer to Table 29).

**Table 29:** Results of standard deviation metric.

Method	N1	N2	N3	N4	N5	N6	N7	N8	N9	N10
Standard deviation	0.1452	0.0593	0.0944	0.1029	0.1117	0.1245	0.0945	0.2151	0.0709	0.0944
Rank	2	10	7	5	4	3	6	1	9	7

However, from the overall perspective, with the aid of the Borda technique, we can conclude N1 is the best fit for TOPSIS, followed by N6 > N3 > N10 > N4 > N8 > N7 > N2 > N5 > N9 (refer to Table 30).

**Table 30:** Final ranks of normalization methods based on the aggregated Borda scores.

Metric/Method	Spearman's rank correlation metric		Pearson correlation metric		Standard deviation metric		Aggregated Borda score	Final rank
	Rank	Borda score	Rank	Borda score	Rank	Borda score		
N1	1	9	2	8	2	8	25	1
N2	6	4	6	4	10	0	8	8
N3	2	8	3	7	7	3	18	3
N4	5	5	5	5	5	5	15	5
N5	10	0	10	0	4	6	6	9
N6	4	6	1	9	3	7	22	2
N7	8	2	7	3	6	4	9	7
N8	9	1	8	2	1	9	12	6
N9	7	3	9	1	9	1	5	10
N10	2	8	3	7	7	3	18	3

## Discussion

As predicted, Table 26 and Fig. 2 prove that employing different normalization methods in TOPSIS results in distinct alternative scores and ranks. This finding does not only apply to TOPSIS but also to other MCDM techniques. For example, Ersoy [44], Vafaei et al. [41], Palczewski and Sałabun [39], and Mathew et al. [37]  reported varying alternative scores and ranks after testing a different combination of normalization methods with ROV, AHP, PROMETHEE, and WASPAS technique, respectively. Interestingly, it can be noticed that, in our study, all normalization methods combined with TOPSIS, except N5 and N8, identified A29 as the least preferred alternative. Based on the TOPSIS principle, this finding interprets that A29 has the shortest geometric distance from the positive-ideal solutions and the farthest distance from the negative-ideal solutions.

The difference in the alternative scores and ranks urged us to further our investigation to identify the most reliable results or, in other words, to trace the most suitable normalization methods for TOPSIS. Hence, we used three metrics to evaluate the suitability of these methods. The evaluation based on the first metric, average Spearman's rank correlation, suggests N1, N2, N3, N4, N6, and N10 as the more suitable methods for TOPSIS. It is because these six normalization methods delivered identical alternative ranks, making them record the highest average Spearman's rank correlation [71]. Jafaryeganeh et al. [42] reported a similar finding where they concluded  N3 and N6 as the ideal choices for TOPSIS after detecting the presence of a strong Spearman's rank correlation in ranks produced by both methods. On the other hand, N5 was detected as the least suitable one since the ranks the method produced were far inconsistent from others, which led to the lowest average Spearman's rank correlation.

The analysis was then extended based on the second metric, hoping to bring more distinction in the suitability rank of the normalization methods. Moreover, the first metric only inspects suitability in the context of alternative ranks and not alternative scores [54]. Thus, the second metric, i.e., average Pearson correlation, was applied. N6 records the highest average Pearson correlation, and therefore it is considered the most suitable method since it delivers alternative scores that agree well with other methods. On the contrary, N5 exhibits the poorest suitability mainly because the alternative scores computed via the method show a strong nonconformity against N8 and N9, with a negative Pearson correlation value, respectively [72]. This finding conforms with the investigation carried out by Chatterjee and Chakraborty [33], in which N6 was declared a more suitable normalization method for TOPSIS compared to N7 and N10.

All the methods were further evaluated based on the third metric, i.e., the standard deviation, to make a more inclusive judgement. This metric was used to evaluate the methods’ ability to spread distinctive scores across the most and least suitable alternatives [40]. Interestingly, N8, which failed to perform well over the previous two metrics, delivered outstanding performance on the third metric, leaving N1 and N6 in the second and third positions, respectively. The method managed to discriminate the preference over the alternatives with distinctive scores. Also, it is surprising that N1 outperforms TOPSIS's default method, N6, on the third metric, indicating that the alternative scores estimated by N6 are not distinct enough. A similar finding was also reported in the literature for other MCDM techniques, confirming its incompetency in producing distinguishing alternative scores. For example, Vafaei et al. [40], who tested the effect of multiple normalization methods on a dynamic MCDM technique, found that N6 recorded the lowest standard deviation compared to N1 and N2.

In also interesting to witness that N3 and N10 share the same suitability rank with respect to all three metrics. Although both methods resulted in different sets of normalized data values, they had the same positive-ideal and negative-ideal solutions for each criterion, thus leading to identical alternative scores and ranks. This discovery leads to two important conclusions. Firstly, we can conclude that N3 and N10 have the same effect on the results of TOPSIS. Secondly, we can also conclude that future comparative studies on TOPSIS can merely incorporate either N3 or N10; it is pointless to include both methods in such comparative studies since both lead to identical alternative scores. This unique discovery might only apply to TOPSIS and not other MCDM techniques.

Since the normalization methods are tagged with different ranks across all three evaluation metrics, it was difficult to conclude which methods suited TOPSIS well. Thus, the analysis was continued by aggregating these ranks using the Borda technique. The computed Borda scores finally suggest N1 as the most suitable normalization method for TOPSIS. If we used the usual plurality voting method to aggregate the ranks, the result would have equally favoured N1, N6, and N8 since each exhibits top-notch performance, i.e. 1st rank, with at least one evaluation metric. It is indeed insensible to equally treat N6 or N8 with N1, mainly because of N6’s and N8’s relatively lower performance against the average Spearman's rank correlation metric. In addition, N8’s performance against the average Pearson correlation metric is abysmal, i.e. 8th rank. However, with the aid of the Borda technique, N1, which holds a decent performance against all three metrics, is selected as the most suitable method. At the same time, it is not surprising to conclude N9 is the least suitable choice for TOPSIS due to its poor performance with respect to all three metrics. In short, the Borda technique's ability to control information loss by mathematically capturing the complete relative rank information across all three metrics has led to this compelling result [73,74].

Another unique finding of this study is that the methods securing the overall 1st  and 2nd  ranks belong to two distinct groups of normalization methods, i.e. the linear and non-linear methods. N1, the method in the 1st rank, belongs to the former, and N6, ranked 2nd, belongs to the latter. Note that both linear and non-linear methods have their own advantage. Linear normalization is usually preferred if the data are distributed evenly across the range of values, but non-linear normalization may be necessary if the data are skewed or if there are outliers. Thus, this study recommends that TOPSIS users should first analyze the distribution of the data in the decision matrix before choosing the suitable normalization method. If the data are evenly distributed, they can opt for the best linear normalization method identified in this study, i.e.,  N1. Otherwise, N6, the best non-linear method, can be applied.

However, for safer decision-making, this study strongly recommends that users consider combining N1 and N6 into the TOPSIS application. They are encouraged to aggregate the ranks from both methods before making decisions based on the TOPSIS results. Milani et al. [27] and Zavadskas et al.[75]  also made a similar recommendation. Still, unlike our study, they did not carry out a thorough investigation to suggest the exact ideal combination of a linear and non-linear normalization method for TOPSIS.

## Conclusion

Literature reports that the choice of normalization methods can affect the results produced by an MCDM technique. Numerous studies have thus been conducted to compare and recommend suitable normalization methods for an MCDM technique, including TOPSIS. However, the existing TOPSIS-related studies either tested a limited number of normalization methods or used incomprehensive metrics to evaluate the methods’ suitability. As such, the conclusiveness of the previous studies can be called into question, leaving the TOPSIS users in the dilemma of whether to adhere to the normalization methods recommended in those studies. In light of these limitations, this study decided to employ an alternate, comprehensive procedure to evaluate and recommend suitable BCC-based normalization methods for TOPSIS (out of ten methods traced from past literature). The procedure was devised using three evaluation metrics, namely the average Spearman's rank correlation, the average Pearson correlation, and the standard deviation metrics, merged with the Borda count technique.

The results suggest that TOPSIS users must analyze the data distribution in the decision matrix before choosing the suitable normalization method. If the data are evenly distributed, they can opt for the best linear normalization method identified in this study, the sum-based method. Otherwise, the vector method, the finest non-linear method, can be applied. This study, however, strongly suggests that users should consider incorporating the sum-based method and vector method into TOPSIS. They can aggregate the ranks from both methods before making decisions based on the TOPSIS results.

The study also concludes the enhanced accuracy method is the least suitable choice for TOPSIS due to its inefficiency in producing reliable alternative ranks or failure to spread distinctive scores across the least or most preferred alternatives. Also, the study discovered that applying the maximum method (version I) or Jüttler's-Körth's method does not cause any difference to the TOPSIS results. Although both methods generate different normalized data sets, they result in the same positive-ideal and negative-ideal solutions, thus leading to identical alternative scores and ranks.

From the literature viewpoint, this study can be regarded as the first to consider a total of ten BCC-based normalization methods to offer more concrete recommendations to address the ongoing arguments about the suitable normalization methods for TOPSIS. Past investigations indeed only tested a maximum of six normalization methods.

Meanwhile, from the methodological point of view, this is the first comparative study that used a combination of three different evaluation metrics with the Borda technique to guarantee comprehensiveness and minimize possible bias in selecting ideal normalization methods for TOPSIS. Using three evaluation metrics enabled us to test the suitability of all ten normalization methods based on crucial perspectives. To be exact, the methods were tested based on their ability to produce reliable alternative scores, generate reliable alternative ranks and spread distinctive scores across the most and least preferred alternatives. Whereas the use of the Borda technique ensured the normalization method that has a decent performance across all three metrics is selected as the ideal method.

On the other hand, from the practical standpoint, this study conveys solid recommendations for users to consider merging both the sum-based method and vector method into the TOPSIS application for a more reliable result. In other words, they should aggregate the ranks from both methods to enable safer decision-making. The conclusive recommendations reported in this study could also help users feel surer that they are employing the best possible normalization methods, thus enabling the decisions based on TOPSIS results to be executed with better confidence.

Nevertheless, this study has a limitation. It merely traced and compared the suitability of the BCC-based normalization methods over TOPSIS. The study's scope was narrowed to BCC-based methods on the ground that they can consider whether a criterion is a benefit or cost-oriented criterion before normalization, thus permitting a logical comparison of an alternative across different criteria. The non-BCC-based methods, e.g., the z-score normalization, the decimal normalization, reference-based normalization etc., were sidelined since they are just appropriate for a homogenous decision matrix, which does not contain a mixture of benefit or cost criteria. However, a future comparative study may also broaden its attention to recommend suitable non-BCC-based methods for TOPSIS, where such a recommendation is helpful in case the MCDM problem at hand only incorporates criteria with parallel orientation*.*

## Supplementary material *and/or* additional information [OPTIONAL]

Not applicable.

## Ethics statements

Not applicable.

## CRediT authorship contribution statement

**Anath Rau Krishnan:** Conceptualization, Formal analysis, Methodology, Investigation, Writing – original draft, Visualization. **Mohamad Rizal Hamid:** Validation. **Geoffrey Harvey Tanakinjal:** Writing – review & editing. **Mohammad Fadhli Asli:** Software. **Bonaventure Boniface:** Software. **Mohd Fahmi Ghazali:** Writing – review & editing.

## Declaration of Competing Interest

The authors declare that they have no known competing financial interests or personal relationships that could have appeared to influence the work reported in this paper.

## Data Availability

The data used for the investigation are provided within the manuscript.
